# Hitting Two Birds With One Stone: Dual Modulation of Brain Carbonic Anhydrases and Histone Deacetylases Boosts Memory Consolidation

**DOI:** 10.1002/ardp.70020

**Published:** 2025-06-18

**Authors:** Alessia Costa, Murat Bozdag, Gioele Renzi, Barbara Rani, Maria Beatrice Passani, Andrea Angeli, Gustavo Provensi, Fabrizio Carta, Claudiu T. Supuran

**Affiliations:** ^1^ Department of NEUROFARBA, Section of Pharmacology and Toxicology, Laboratory of Ocular and Neuropsychopharmacology (Braeye Lab) University of Florence Florence Italy; ^2^ NEUROFARBA Department, Pharmaceutical and Nutraceutical Section University of Florence Florence Italy; ^3^ Department of Health Sciences, Laboratory of Ocular and Neuropsychopharmacology (Braeye Lab) University of Florence Florence Italy

**Keywords:** carbonic anhydrase activators, cognition, d‐phenylalanine, histone deacetylase inhibitors, metalloenzymes, multitarget ligands

## Abstract

Cognitive impairments, characterized by deficits in one or more cognitive domains, are common in several pathological conditions but remain inadequately addressed by available pharmacological treatments. Given the complexity and multifaceted mechanisms underpinning these deficits, multi‐targeted directed ligands are emerging as promising strategies for developing more effective therapies. In this study, we reported the design, synthesis as well as In Vitro and Ex Vivo assessment of prototypic molecular scaffolds that activate the carbonic anhydrase (CA; EC 4.2.1.1) and inhibit the histone deacetylase (HDAC; EC 3.5.1.98) metalloenzymes. By using the novel object recognition paradigm, we found that these compounds significantly enhanced memory consolidation at doses 10 times lower than single‐target reference compounds. Taken together, these results suggest that the dual modulation of CA and HDAC activities by means of a single hybrid molecular entity represents an innovative approach for the management of cognitive symptoms associated with neurodegenerative, neurodevelopment, and psychiatric disorders.

## Graphical abstract

Multitarget‐directed ligands were obtained by merging the carbonic anhydrase (CA) activating histamine with the selective histone deacetylase (HDAC) inhibitor of the benzamide type. The derivatives showed good CA activation and HDAC inhibition In Vitro and Ex Vivo, and enhanced recognition memory consolidation at doses 10‐fold lower than the single reference drugs.

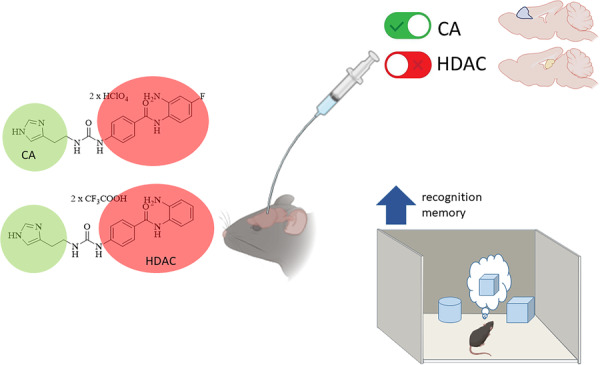

## 1 Introduction

Cognition refers to a complex set of mental processes by which individuals acquire, understand, process, and apply information. It encompasses a broad range of functions including perception, attention, memory, reasoning, language, and executive functions such as problem‐solving, decision‐making, and planning. Healthy cognitive functioning enables individuals to learn from experiences, adapt to new situations, and produce appropriate responses to the environment by shaping the way they think, feel, and behave [1]. When these functions are impaired, a series of symptoms may appear ranging from mild difficulties in concentrating, in understanding language, or in organizing and completing tasks, confusion, and memory lapses in early stages until disorientation, inability to recognize familiar people or places, and severe memory loss in advanced stages [2, 3]. These symptoms are associated with different disorders, the most common ones being neurodegenerative diseases such as Alzheimer's disease, Parkinson's disease, and multiple sclerosis, where progressive damage to brain cells leads to a decline in memory, attention, and reasoning abilities [3]. Traumatic brain injury and stroke can also cause sudden cognitive symptoms, including memory loss, confusion, and difficulty of concentrating, as they disrupt normal brain function [4]. Additionally, psychiatric disorders such as depression, anxiety, and schizophrenia can impair cognitive functions, particularly attention and memory [5]. Other chronic medical conditions such as diabetes, hypertension, or heart disease can affect cognitive performance, especially if they result in reduced blood flow to the brain [6]. Substance abuse, including alcohol, may also lead to cognitive deficits over time [7]. Improving cognitive functioning is a recognized goal of treatment for all these conditions [8].

Cognitive symptoms are often the result of complex and interconnected pathological processes, such as neurotransmitter imbalances, oxidative stress, neuroinflammation, and neurodegeneration [9, 10]. Thus, single‐target therapies may fail to address their multifaceted nature, leading to limited efficacy in improving cognitive function or halting disease progression. In this context, multi‐target directed ligands (MTDLs) are emerging as a new promising approach by simultaneously modulating multiple pathways, offering a more comprehensive therapeutic strategy to improve treatment outcomes while reducing the need for polypharmacy and its associated risks, such as drug interactions and side effects [11, 12]. Capitalizing on these concepts, here we aimed at developing novel compounds targeting relevant metalloenzymes critically involved in cognitive functions such as the carbonic anhydrases (CAs, EC 4.2.1.1) and histone deacetylases (HDAC, EC 3.5.1.98).

Epigenetic mechanisms, including posttranslational modifications of histones, exert dynamic control over cognitive functions [13]. For example, enhanced removal of acetyl groups from histones, mediated by HDACs, leads to chromatin condensation and transcriptional repression, which in turn results in suppression of the expression of genes critically involved in learning and memory [14]. Consistently, multiple studies demonstrated the efficacy of treatment with different HDAC inhibitors as memory enhancers in both healthy and cognitively impaired animals [15, 16, 17]. FDA‐approved HDAC inhibitors (e.g., vorinostat and valproic acid) are being explored for cognitive enhancement in neurodegenerative and psychiatric conditions [18]. CAs are ubiquitously expressed enzymes that reversibly catalyze the conversion of carbon dioxide and water into bicarbonate and protons. Such reaction is critically involved in pH balance, gas exchange, and ion transport [19]. Beyond the CAs physiological roles in respiration and acid–base homeostasis, growing evidence indicates that modulation of brain CAs activity has bidirectional effects on animal learning [20]. For instance, CAs inhibition impaired, whereas CAs activation facilitated memory consolidation in different tasks such as the Morris water maze [21], shuttle and passive avoidance [22], novel object recognition [23], contextual fear extinction [24], and social discrimination [25].

In this study, we describe the design and synthesis of prototypic MTDLs endowed with remarkable CA activating and strong HDAC inhibiting features In Vitro and validated their action Ex Vivo on brain area specimens critically involved in the stabilization of mnemonic traces. Consistently, when evaluated In Vivo, these compounds enhanced recognition memory consolidation in doses significantly lower than the reference compounds, suggesting that the dual modulation of CA and HDAC enzymes is a promising strategy for treating cognitive deficits associated with different pathological conditions.

## 2 Results and Discussion

### 2.1 Design and Synthesis

**Figure 1:**
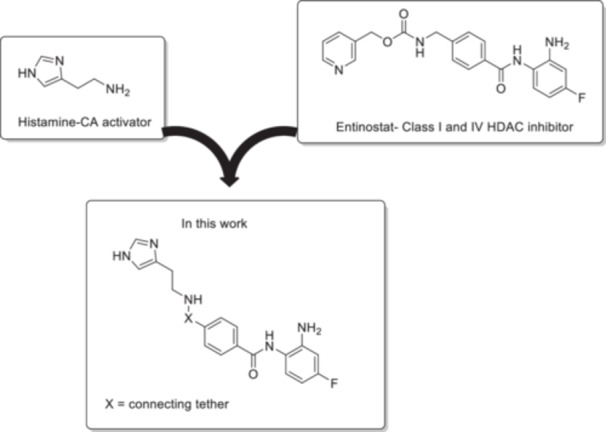
Schematic representation of the design strategy adopted.

For the purposes of this study, we envisage to merge within a single molecular entity the prototypic CA activating moiety histamine with a selective HDAC inhibitory moiety of the benzamide type as schematically reported in Figure 1.

**Scheme 1:**
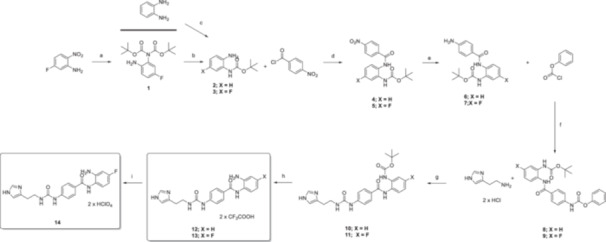
Synthesis of final compounds **12** and **14**. Reagents and conditions: (a) Boc_2_O (2.0 eq.), Et_3_N (3.0 eq.), DMAP (0.01 eq.), DCM, o.n.; CH_3_COOH (0.6 eq.), H_2_O/EtOH (2/1), r.t., 2 h. (b) CF_3_COOH (1.0 eq.), DCM, 0°C–r.t., 1 h. (c) Boc_2_O (1.0 eq.), 1.0 M aq. NaOH (1.0 eq.), DCM, r.t., o.n. (d) Et_3_N (1.5 eq.) in DCM, 0°C–r.t., 4 h. (e) Fe (0) powder (6 eq.), CH_3_COOH (0.6 eq.), H_2_O/EtOH (2/1), r.t. – 70°C, 4 h. (f) DIPEA (1.2 eq.), THF, 0°C–r.t., 4 h. (g) Et_3_N (2.0 eq.), ACN, 80°C, o.n. (h) CF_3_COOH (5.0 eq.), DCM, 0°C – r.t., 2‐3 h. (i) NaClO_4_ (5.0 eq.), H_2_O, 55°C – r.t.

Our interest in benzamides are ascribed to the high selectivity of this moiety for HDACs both In Vitro and In Vivo, thus with very limited interferences with other metalloenzymes, which typically are very abundant in human tissues, such as the CAs [26, 27]. The first successful application of this kind was afforded by the HDAC inhibitor Entinostat currently under advanced clinical stage of investigation as an anticancer therapeutic agent by promoting histone hyperacetylation and transcriptional activation of specific genes [26, 27, 28, 29]. In addition, the subclass selectivity of Entinostat for class I and IV HDACs (i.e., HDAC isozymes 1–3, 8, and 11) is ensured by the pyridine recognition cap, which is engaged in specific interactions with the apoenzymes [29]. In line with such design planning, we synthesized the final compounds **12** and **14** as reported in Scheme 1.

The multistep synthesis toward the desired compounds started with regioselective monoprotection of the commercially available benzene‐1,2‐diamine to afford **2**, whereas its fluoro derivative **3** was obtained by full protection of 5‐fluoro‐2‐nitroaniline followed by reduction of the nitro moiety. Both intermediates were subjected to the same reaction procedures as in Scheme 1, which accounted for the derivatization of the free anilines with 4‐nitrobenzoyl chloride, nitro groups reduction in **4** and **5** followed by reaction with phenyl chloroformate to introduce a specific point of attack for the nucleophilic histamine. Removal of the Boc protection in **10** and **11** afforded the final compounds **12** and **13** as trifluoroacetic salts. Although no differences were reported for the chemistry and manipulation of the two‐compound series through all the synthetic steps in Scheme 1, the fluoro derivative **13** proved tedious to manipulate and hard to purify up to an acceptable grade for In Vivo use. We solved the problem by switching the trifluoroacetic acid in **13** with the biologically inactive perchlorate instead, to afford **14** as a crystalline and satisfactory pure precipitate in high yields (Scheme 1).

**Scheme 2:**
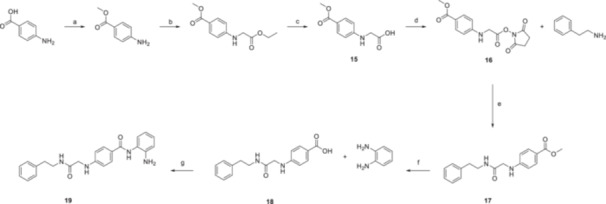
Synthesis of **19**. Reagents and conditions: (a) SOCl_2_ (5.0 eq.), MeOH, 0°C–r.t., 4 h. (b) Ethyl chloroacetate (1.1 eq.), K_2_CO_3_ (2.0 eq.), DMA, 75°C, 2 h. (c) 5% aq. NaOH (1.1 eq.), EtOH, r.t. 5 min. (d) NHS (1.5 eq.), EDCI (1.5 eq.), DMA, 0°C – r.t., 2 h. (e) Et_3_N (2.0 eq.), DMA, r.t., on. (f) LiOH (5.0 eq.), H_2_O/THF/MeOH (1/2/2), r.t., on. (g) HOAt (1.5 eq.), EDCI (2.0 eq.), Et_3_N (4.0 eq.), DMA, 0°C–r.t., 4 h.

To assess the target specificity of **12** and **14**, we sought to make use of their related molecules having the CA activating and HDAC inhibiting moieties alternatively silenced. Specifically, **10** in Scheme 1 is devoid of the HDAC targeting moiety, whereas the derivative **19**, synthesized in agreement with the Scheme 2, is deprived of the imidazolyl activation head for the CA counterparts.

The linear synthetic approach to **19** began with the protection of the commercially available 4‐amino benzoic acid as methyl ester, insertion of the protected glycine, and its cleavage to afford **15**. Then the free carboxylic acidic moiety was converted to the super ester **16**, which in turn was readily exposed to nucleophilic attach from phenethylamine allowing to insert the CA ineffective head in **17**. The final compound **19** was successfully obtained by installation of the HDAC benzamidic‐directed moiety by coupling of **18** with benzene‐1,2‐diamine (Scheme 2). All the final compounds herein reported were purified by silica gel column chromatography using the appropriate eluting mixtures and followed by trituration or recrystallization as needed (see Section 4). Full characterization was conducted by means of solution ^1^H‐ and ^13^C‐NMR. Elemental analyses account for a purity grade ≥ 96%.

### 2.2 In Vitro CA Profiling

The impact of compounds **12**, **14**, and **19** on the catalytic activity of physiologically relevant human (h) CAs isoforms was assessed In Vitro through the stopped‐flow CO_2_ hydrase assay [30]. The potencies were reported as *K*_A_ values in Table 1 and compared with the reference compound d‐Phe. AC_50_ curves are reported in the Supporting Information.

**Table 1:** Activation data of compounds 12, 14, and 19 compared with reference d‐Phe on hCAs I, II, IV, VA, VII, IX were obtained through the stopped‐flow CO_2_ hydrase assay [30] and are expressed as the *K*_A_ values.

*K*_A_ (µM)^a^						
Compounds	hCA I	hCA II	hCA IV	hCA VA	hCA VII	hCA IX
**12**	75.4	66.8	23.0	> 10000	32.0	68.8
**14**	53.8	49.5	49.1	37.9	26.1	> 10000
**19**	> 10000	> 10000	> 10000	> 10000	> 10000	> 10000
d‐Phe	86.0	0.035	49.3	4.63	9.74	9.3

^a^ Mean from three different assays, by the stopped‐flow CO_2_ hydrase assay (errors were in the range of ±5%–10% of the reported values).

Compound **19** is an ineffective modulator (i.e., *K*_A_ > 10000 µM) on the panel of hCAs considered in this study, thus demonstrating that imidazolyl moiety is essential for activation of the CAs [31]. Of note, the HDAC targeting benzamidic moiety does not interfere with the catalytic activity of such enzymes, thus making this compound as suitable control acting solely on HDAC enzymes.

Distinct CA activation profiles were reported for compounds **12** and **14**. Such differences are undoubtedly ascribed to the substitution of a proton in **12** with a fluorine atom in **14**. To the best of our knowledge, this is the first example of halogen‐dependent kinetic modulation for CA activators, whereas such properties are widely described for CAIs [32]. Compounds **12** and **14** were ineffective (i.e., *K*_A_ > 10000 µM) on the mitochondrial expressed hCA VA and on the tumor‐associated hCA IX isoforms, respectively. Small *K*_A_ value differences were found for the other isoforms with **14** being a potent activator of hCAs I (i.e., 1.4‐fold), II (i.e., 1.3‐fold), and VII (i.e., 1.2‐fold). For the purposes of this study, the *K*_A_s of **12** and **14** associated with the abundantly central nervous system (CNS) expressed hCA VII are of relevance since are almost matchable.

### 2.3 In Vitro HDAC Profiling

**Figure 2:**
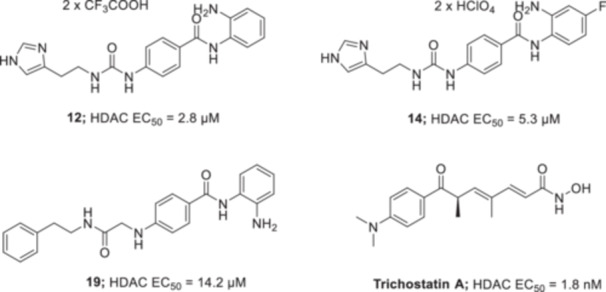
Structures and EC_50_ values of the benzamide HDACIs **12**, **14**, and **19** synthesized within this article.

Compounds **12**, **14**, and **19** (Figure 2) were assessed as HDAC activity inhibitors against HeLa nuclear extract by using Fluor de Lys‐Green HDAC assay kit. The potencies were reported as EC_50_ values and compared with the reference compound Trichostatin A. Obtained concentration are mean from three different assays (errors were in the range of 5%–10% of the reported values). EC_50_ curves are reported in the Supporting Information.

Trichostatin A exhibited potent inhibition of the HDAC enzyme (EC_50_ = 1.8 nM), experimentally in agreement with reported literature [33]. On the other hand, the HDAC targeting benzamidic compounds showed lower inhibitory potency with EC_50_ values ranging from 2.8 to 14.2 µM. Compounds **12** and **14** bearing the imidazolyl moiety, essential for CAs activation, showed a better inhibition profile, with EC_50_ = 2.8 µM and EC_50_ = 5.3 µM, respectively. Interestingly, the proton substitution of **12** with a fluorine **14** atom led to diminished activity, likely ascribed to the electron‐withdrawing features of the halogen. In contrast, compound **19** showed a higher effective concentration with EC_50_ = 14.2 µM. However, all benzamidic compounds highlighted a worse inhibitory profile despite exhibiting preferential selectivity.

### 2.4 Ex Vivo Measurement of Brain CA and HDAC Enzymatic Activity

The impact of treatment with the compounds **12**, **14**, **19**, and d‐Phe on CA and HDAC activities was evaluated in two brain regions critically involved in memory processes: hippocampus and frontal cortex. Specifically, mice were euthanized 30 min after the i.c.v. infusion, their brains were collected, and hippocampi and frontal cortices were immediately dissected on ice and then individually homogenized in 1 mL of ice‐cold 20 mM Hepes buffer (pH 7.5). Controls received a comparable injection of vehicle, and the brain samples were collected and processed in the same way.

**Figure 3:**
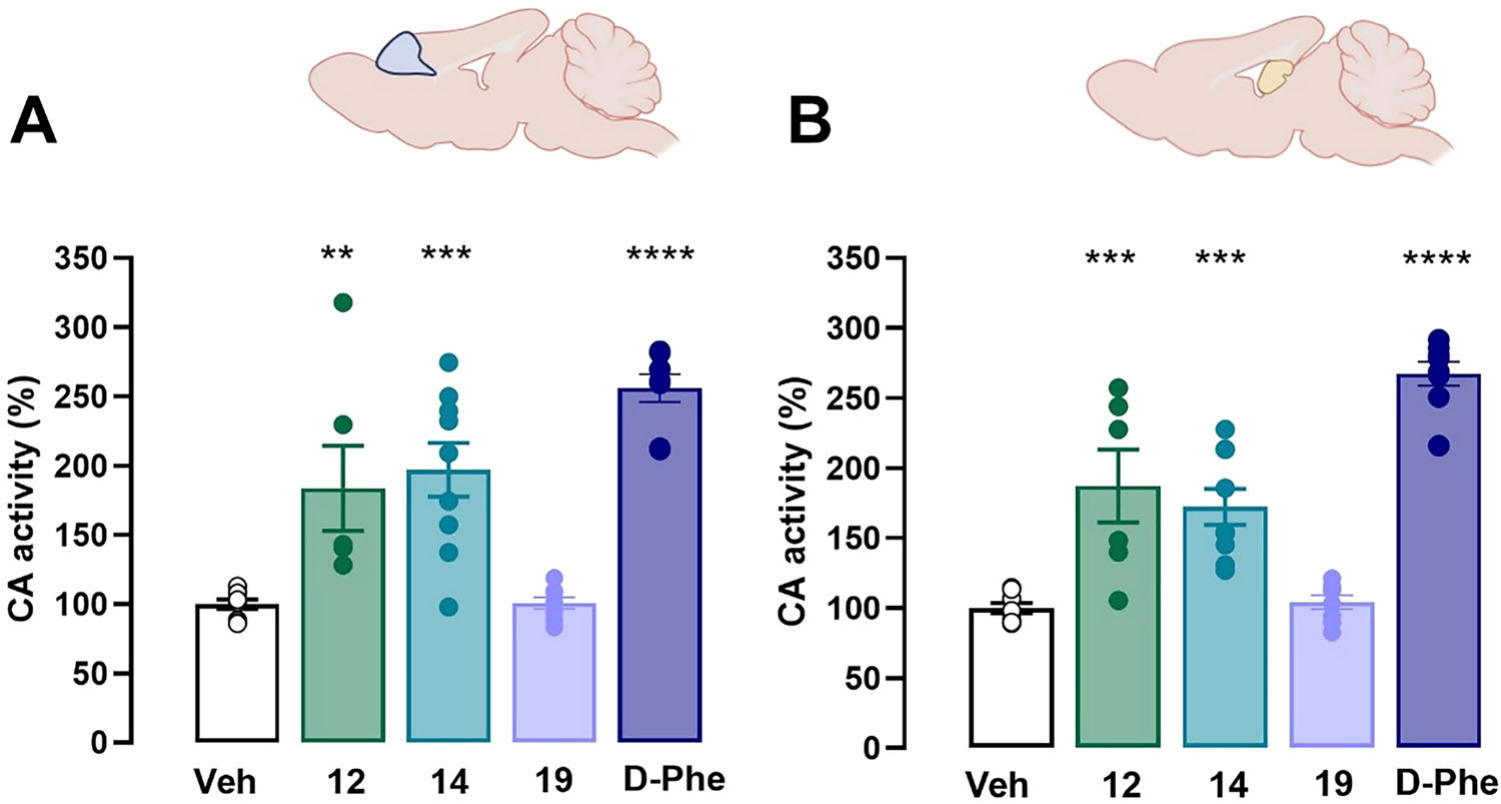
CA activity (%) measured in cortical (A) and hippocampal (B) brain homogenates obtained from mice treated with vehicle or compounds **12** and **14** (50 nmol/µL, i.c.v.). Animals were killed 30 min after drug infusion, and the brain structures were immediately dissected on ice and homogenized. CA activity was measured with a stopped‐flow method. The mean CA activity measured in the group of control mice treated with vehicle was taken as 100%, the activities measured for animals treated with compounds were normalized to this value. Data are expressed as means ± S.E.M. of six–nine mice per experimental group. One‐way ANOVA and Bonferroni MCT, **p < 0.01 versus vehicle.

The overall catalytic activity of CAs (*K*_cat_) of each individual preparation was measured with a stopped flow spectrophotometric method monitoring the physiological CO_2_ hydration reaction catalyzed by these enzymes [30] (Figure 3). Treatments with **12**, **14**, and **d‐Phe** significantly increased the CA activity in mice cortical (*F*_2,21_ = 8,878, *p* < 0.001) and hippocampal (*F*_2,20_ = 9,989, *p* < 0.001) homogenates compared with controls. Noteworthy, no significant differences emerged between the compounds in terms of CA activity in both regions investigated. Such a trend may be ascribed to the equipotent In Vitro activating profile of **12** and **14** on hCA VII along with the comparable effectiveness in engaging the cooperative isoforms hCAs I and II.

Consistent with In Vitro data indicating that compound **19**, at concentrations up to 10.000 µM, did not alter the activity of selected CA isoforms, no differences were observed in CA activity measured Ex Vivo in cortical and hippocampal homogenates from animals treated with compound **19** compared with those treated with the vehicle.

**Figure 4:**
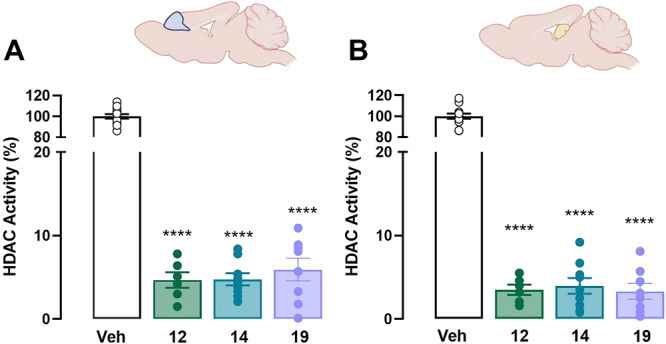
HDAC residual activity (%) measured in hippocampal (A) and cortical (B) brain homogenates obtained from mice treated with vehicle or **12**, **14**, and **19** (50 nmol/μL, i.c.v.). Animals were killed 30 min after drug infusion, and the brain structures were immediately dissected on ice and homogenized. HDAC activity was measured with the fluorometric method. The HDAC mean activity of the control group mice was taken as 100%, % of the activities measured for animals treated with compounds were normalized to this value. Data are expressed as means ± S.E.M. of 6–13 mice per experimental group. One‐way ANOVA and Bonferroni MCT, ****p < 0.0001 versus vehicle‐treated group.

Freshly obtained hippocampal and cortical samples were also used to assess the effects on HDAC activity following treatments with **12** and **14**. The overall residual catalytic activity of HDACs (*K*_cat_) for each individual preparation was assessed through a fluorometric method monitoring the physiological reaction catalyzed by such enzymes (Figure 4).

Overall, the obtained data showed marked differences in residual HDAC activity measured in both the cortex (*F*_4,37_ = 747.8, *p* < 0.0001) and hippocampus (*F*_4,37_ = 713.3, *p* < 0.0001) across the experimental groups. Significant differences were observed between the control group treated with vehicle and the groups receiving compounds **12** and **14**. The selective benzamidic HDAC inhibitor **19** (*p* < 0.0001) determined a significant reduction of enzymatic activities in both regions. Similar effects were observed following the treatment with dual acting compounds **12** (*p* < 0.0001) and **14** (*p* < 0.0001).

### 2.5 Impact of Single‐ and Dual‐Modulation of CA and HDACs on Recognition Memory

This set of experiments was designed to evaluate the impact of single reference compounds (d‐Phe and **19**) as well as the dual acting (**12** and **14**) on recognition memory. These compounds were administered directly into the brain lateral ventricles immediately after the acquisition session to investigate their impact specifically on memory consolidation. The intertrial interval of 24 h was chosen to exploit the “natural” forgetting (Figure 4A) since in previous studies we found that control animals do not remember familiar stimuli at this time point [34, 35]. Such a design avoids the use of amnestic drugs that could interfere with data interpretation and represents an advantage when studying the procognitive potential of novel compounds. The dose range and administration route were also chosen based on previous reports showing that the procognitive effects of CA and HDAC modulators rely on their action on centrally and not peripherally expressed enzymes [23, 36]. Moreover, the injection of the tested compounds directly into the lateral ventricles (i.c.v.) consents widespread drug distribution in the brain and at the same time minimizes potential interference of peripheral phenomena such as barriers crossing, protein binding, drug metabolism, that are highly influenced by specific drug characteristics such as size, solubility, charge. Therefore, the i.c.v. route represents an advantage in studies comparing the potency of compounds by ensuring precise control of drug concentrations in the target organ and excluding confounding factors related to peripheral pharmacokinetic mechanisms [37].

**Figure 5:**
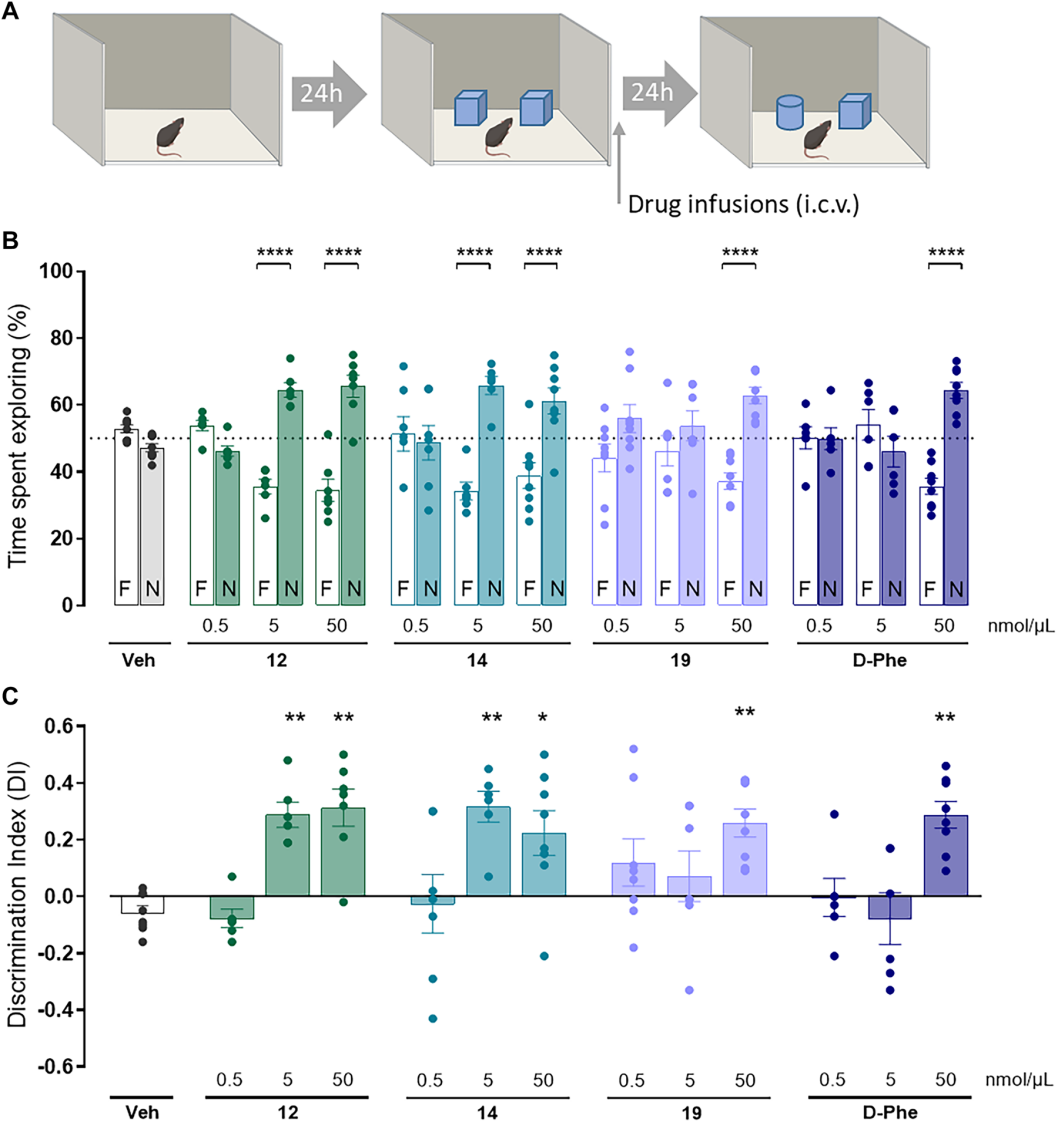
Dual modulation of CAs and HDACs potently boost recognition memory in mice. (A) Schematic drawing showing the sequence of the behavioral procedures: immediately after the acquisition phase mice received infusions (5 µL) of solutions containing either vehicle, the CA activator d‐Phe, the HDAC inhibitor **19** dual acting compounds **12** and **14** at 0.5, 5, or 50 nmol/µL concentrations. The retention test was performed 24 h later. (B) Percentages of time spent exploring the familiar (F) or novel (N) objects during the retention test. The dashed line indicates a theoretical means of 50%. Statistically significant differences determined by two‐way ANOVA and Bonferroni's MCT: ****p < 0.0001 F versus N. (C) Discrimination indexes calculated with the formula *DI* = (*tN tF*)/(*tN* + *tF*). Statistically significant differences determined by one‐way ANOVA and Bonferroni's MCT: *p < 0.05; **p < 0.01 versus vehicle‐treated group. In both graphics, data are expressed either as individual data points for each animal (scattered plot) znc means ± SEM represented by bar graphs. *N* = 6–9 animals by experimental group.

The performances of animals treated with **12**, **14**, **19**, and d‐Phe in the novel object recognition paradigm are shown in Figure 5B. Two‐way ANOVA revealed significant differences between experimental groups in terms of percentage of time spent exploring the familiar and novel objects during the retention test (*F*_objects (1,156)_ = 86.17, *p* < 0.0001; F_treatments (12,156)_ = 2.3e–0.1, *p* > 0.9999, *F*_interaction (12, 156)_ = 10.59, *p* < 0.0001, Figure 5B). Under the conditions used in the present study, the animals pertaining to the control group (treated with vehicle) were unable to recognize the object previously presented during the acquisition phase as revealed by the similar percentage of time they spent exploring familiar and novel objects during the retention test. On the contrary, dose‐dependent effects were observed in the groups of animals treated with the new compounds. At the lowest dose (0.5 nmol/μL), none of the compounds affected the animals’ memory, as they behaved similarly to animals receiving infusions of vehicle, that is, they did not discriminate between familiar and novel objects. Of note, mice that received infusions of compounds **12**, **14** at the doses of 5 and 50 nmol/μL spent significantly more time exploring the novel object (**12**, *p* < 0.0001; **14**, *p* < 0.0001). The promnesic effect of compounds d‐Phe and **19** was achieved only at the highest dose (d‐Phe, *p* < 0.0001; **19**, *p* > 0.0001).

The analysis of the discrimination indexes (DIs, Figure 5C) largely confirmed the observations described above. One‐way ANOVA revealed overall statistically significant differences between groups (*F*_15,78_ = 5.332, *p* < 0.001). The DIs calculated for the animals receiving infusions of compounds at the 50 nmol/µL concentration were significantly higher (**12**: 0.313 ± 0.173, *p* < 0.01; **14**: 0.224 ± 0.223, *p* < 0.05; **19**: 0.259 ± 0.139, *p* < 0.01; d‐Phe: 0.288 ± 0.132, *p* < 0.01) than the DI calculated for animals pertaining to vehicle‐treated group (Veh: −0.057 ± 0.069). No differences emerged in terms of the DIs calculated for the groups of animals treated with vehicle or the 0.5 nmol/µL dose of each compound (**12**: −0.077 ± 0.078, **14**: −0.026 ± 0.274, **19**: 0.120 ± 0.236; d‐Phe: −0.003 ± 0.164). Significant differences emerged between the groups receiving drug infusions at 5 nmol/µL dose: when compared with the control group, significantly higher DI values were calculated for the groups treated with compounds **12** (0.288 ± 0.110, *p* < 0.01), **14** (0.317 ± 0.132, *p* < 0.01), whereas no differences were found for d‐Phe‐ and **19**‐treated groups (−0.078 ± 0.224 and 0.071 ± 0.237, respectively).

From a therapeutic perspective, the cocktail strategy—combining drugs acting at different targets—is commonly employed to achieve synergistic effects, particularly in the treatment of multifactorial disorders. The findings presented here not only support current evidence regarding the impact of individual modulation of CAs and HDACs activities in memory processing [20, 38] but also expand our previous reports demonstrating the effectiveness of the multitargeting strategy in obtaining potent promnesic compounds [34, 39]. Therefore, dual modulation of these enzymatic systems by means of a single molecular entity is a valuable approach for developing new centrally acting drugs.

## 3 Conclusions

Cognitive impairments are common symptoms of various pathological conditions, yet currently available drugs offer only limited benefits in improving cognitive function. Given the complexity of the mechanisms underlying cognitive deficits, a multitargeting strategy may provide a promising avenue for developing innovative therapies. In this study, we designed and synthesized benzamide derivatives, compounds **12** and **14**, that target two key regulators of memory consolidation: CAs and HDACs. The dual action of these compounds was confirmed through Ex Vivo analyses, which revealed reduced HDAC and enhanced CA enzymatic activities in the hippocampus and frontal cortex—two brain regions critical for memory processing. Consistently, In Vivo studies demonstrated that these compounds exerted potent procognitive effects, effectively reversing memory deficits at doses 10 times lower than those required for single‐target reference drugs. Overall, these findings highlight the potential of dual CA and HDAC modulation as a novel pharmacological strategy to enhance cognitive function.

## 4 Experimental

### 4.1 Chemistry

#### 4.1.1 General Remarks

Anhydrous solvents and all chemical and reagents were purchased from Sigma‐Aldrich (Milan, Italy) Alfa Aesar and Fluorochem and Bio‐Techne s.r.L (Milan). All reactions involving air‐ or moisture‐sensitive compounds were performed under a nitrogen atmosphere using dried glassware and syringes to transfer solutions. Nuclear magnetic resonance (^1^H‐NMR, ^13^C‐NMR, ^19^F‐NMR) spectra (see the Supporting Information) were recorded using a Bruker Avance III 400 MHz spectrometer in DMSO‐*d*_6_. Chemical shifts are reported in parts per million (ppm) and the coupling constants (*J*) are expressed in Hertz (Hz). Splitting patterns are designated as follows: s, singlet; d, doublet; t, triplet; q, quartet; m, multiplet; dd, doublet of doublets; bs, broad singlet; ap s, apparent singlet; ap d, apparent doublet; ap t, apparent triplet; ap q, apparent quartet. The assignment of exchangeable protons (O*H* and NH) was confirmed by the addition of D_2_O. Analytical thin‐layer chromatography (TLC) was carried out on Merck silica gel F‐254 plates. Flash chromatography purifications were performed on Merck silica gel 60 (230‐400 mesh ASTM) as the stationary phase and methanol/dichloromethane (MeOH/DCM) or ethyl acetate/hexane (EtOAc/Hex) were used as eluents. The solvents used in mass spectra (MS) measures were acetone, acetonitrile (Chromasolv grade), and 56 mQ water 18 MΩ, obtained from Millipore's Simplicity system (Milan‐Italy). The MS were obtained using a Varian 1200 L triple quadrupole system (Palo Alto, USA) equipped by an electrospray source (ESI) operating in both positive and negative modes. Stock solutions of analytes were prepared in acetone at 1.0 mg/mL and stored at 4°C. Working solutions of each analyte were freshly prepared by diluting stock solutions in a mixture of mQ H_2_O/ACN 1/1(v/v) up to a concentration of 1.0 µg/mL. The MS of each analyte were acquired by introducing, via syringe pump at 10 L/min, the working solution. RawQdata were collected and processed by Varian Workstation Vers. 6.8.

The InChI codes of the investigated compounds, together with some biological activity data, are provided as Supporting Information.

#### 4.1.2 Synthesis of Bis‐*Tert*‐Butyl (2‐Amino‐4‐Fluorophenyl)Carbamate **1**

To a solution of 5‐fluoro‐2‐nitroaniline (1.0 eq.) in DCM (10 mL) was added Boc_2_O (2.0 eq.), Et_3_N (3.0 eq.), DMAP (0.01 eq.). The mixture was stirred at room temperature overnight, concentrated under vacuum, extracted with DCM, and dried over Na_2_SO_4_. The product was pure enough to be used as it was. White solid. Yield 42%. ^1^H NMR (400 MHz, DMSO‐*d*_6_) *δ* 8.15 (1H, dd, *J* = 5.43, *J* = 9.13 Hz, Ar‐H), 7.19 (1H, m, Ar‐H), 7.05 (1H, dd, *J* = 2.71, *J* = 8.32 Hz, Ar‐H), 1.59 (2H, brs, exchange with D_2_O, –NH_2_), 1.41 (18H, s, C‐(CH_3_)_3_ ×2). The compound is in agreement with reported literature [40].

#### 4.1.3 Synthesis of *Tert*‐Butyl (2‐Aminophenyl)Carbamate **2**

A solution of *o*‐phenylenediamine (1.0 eq.) in DCM (10 mL) was added to a solution of Boc_2_O (1.0 eq.) in 1 M aqueous NaOH (1.0 eq.). The mixture was stirred at room temperature overnight, concentrated under vacuum, extracted with DCM, and dried over Na_2_SO_4_. The product was pure enough to be used as it was. White solid. Yield 94%. ^1^H NMR (400 MHz, DMSO‐*d*_6_) *δ* 8.28 (1H, s, exchange with D_2_O, –NH), 7.17 (1H, d, *J* = 7.99 Hz, Ar‐H), 6.83 (1H, m, Ar‐H), 6.67 (1H, dd, *J* = 1.43, *J* = 7.96 Hz, Ar‐H), 6.51 (1H, m, Ar‐H), 4.81 (2H, s, exchange with D_2_O, ‐NH_2_), 1.45 (9H, s, C‐(CH_3_)_3_); ^13^C NMR (101 MHz, DMSO‐*d*_6_) *δ* 154.5 (C), 142.1 (CH), 125.9 (CH), 124.6 (CH), 117.2(CH), 116.6 (CH), 79.5 (C), 29.1 (CH_3_ x3); *m/z* (ESI positive) 209.12 [M+H]^+^; Elemental analysis calculated (%):C, 63.44; H, 7.74; N, 13.45; found: C, 63.47; H, 7.72; N, 13.43.

#### 4.1.4 Synthesis of *Tert*‐Butyl (2‐Amino‐4‐Fluorophenyl)Carbamate **3**

bis‐*tert*‐Butyl (2‐amino‐4‐fluorophenyl)carbamate **1** (1.0 eq.) was dissolved in DCM (2 mL) at 0°C. Then, CF_3_COOH (1.0 eq.) was added. The temperature was raised until r.t. and the reaction mixture was stirred for 1 h. The mixture was concentrated under vacuum, extracted with DCM, and dried over Na_2_SO_4_. The product was pure enough to be used as it was. White solid. Yield 55%. ^1^H NMR (400 MHz, DMSO‐*d*_6_) *δ* 8.40 (1H, s, exchange with D_2_O, –NH), 7.19 (1H, d, *J* = 10.9 Hz, Ar‐H), 6.65 (2H, dd, *J* = 1.57, *J* = 6.98 Hz, Ar‐H), 4.76 (2H, s, exchange with D_2_O, –NH_2_), 1.46 (9H, s, C‐(CH_3_)_3_); ^13^C NMR (101 MHz, DMSO‐*d*_6_) *δ* 156.0 (C), 145.5 (C), 141.9 (CH), 130.1 (C), 121.3 (CH), 119.0 (CH), 115.4 (CH), 78.5 (C), 29.1 (CH_3_ x3); *m/z* (ESI positive) 227.11 [M+H]^+^; Elemental analysis calculated (%):C, 58.40; H, 6.68; N, 12.38; found: C, 58.43; H, 6.70; N, 12.35.

#### 4.1.5 Synthesis of *Tert*‐Butyl [2‐(4‐Nitrobenzamido)Phenyl]Carbamate 4 and *Tert*‐Butyl (5‐Fluoro‐2‐(4‐Nitrobenzamido)Phenyl)Carbamate **5**

*tert*‐Butyl (2‐aminophenyl)carbamate **2** or *tert*‐butyl (2‐amino‐4‐fluorophenyl)carbamate **3** (1.0 eq.) was dissolved in dry DCM (10 mL), followed by the addition of Et_3_N (1.5 eq.). Then, 4‐nitrobenzoyl chloride (1.1 eq.) was added at 0°C. The temperature was raised until r.t., and the reaction mixture was stirred for 4 h. The mixture was quenched with H_2_O, extracted with DCM, and dried over Na_2_SO_4_. The crude reaction was purified by flash chromatography (EtOAc/Hex 50:50) to afford *tert*‐butyl [2‐(4‐nitrobenzamido)phenyl]carbamate **4** and *tert*‐butyl [5‐fluoro‐2‐(4‐nitrobenzamido)phenyl]carbamate **5**.

Following the above procedure, *tert*‐butyl (2‐aminophenyl)carbamate **2** gave *tert*‐butyl [2‐(4‐nitrobenzamido)phenyl]carbamate **4** as a yellow solid. Yield 63%. ^1^H NMR (400 MHz, DMSO‐*d*_6_) δ 10.16 (1H, s, exchange with D_2_O, –NH), 8.78 (1H, s, exchange with D_2_O, –NH), 8.38 (2H, d, *J* = 8.56 Hz, Ar‐H), 8.22 (2H, d, *J* = 8.48 Hz, Ar‐H), 7.62 (1H, d, *J* = 7.94 Hz, Ar‐H), 7.52 (1H, d, *J* = 7.67 Hz, Ar‐H), 7.23 (1H, t, *J* = 7.42 Hz, Ar‐H), 7.14 (1H, t, *J* = 7.43 Hz, Ar‐H), 1.44 (9H, s, C‐(CH_3_)_3_); ^13^C NMR (101 MHz, DMSO‐*d*_6_) *δ* 164.8 (C), 154.3 (C), 150.2 (C), 141.1 (CH), 133.4 (CH), 130.2 (C), 129.9 (CH), 127.5 (CH), 127.0 (CH), 124.8 (C), 124.5 (C), 80.5 (C), 46.4 (CH), 29.0 (CH_3_ x3); *m/z* (ESI positive) 358.13 [M+H]^+^; Elemental analysis calculated (%):C, 60.50; H, 5.36; N, 11.76; found: C, 60.47; H, 5.38; N, 11.75.

Following the above procedure, *tert*‐butyl (2‐amino‐4‐fluorophenyl)carbamate **3** gave *tert*‐butyl [5‐fluoro‐2‐(4‐nitrobenzamido)phenyl]carbamate **5** as a yellow solid. Yield 57%. ^1^H NMR (400 MHz, DMSO‐*d*_6_) *δ* 10.02 (1H, s, exchange with D_2_O, –NH), 8.88 (1H, s, exchange with D_2_O, –NH), 8.38 (2H, d, *J* = 8.81 Hz, Ar‐H), 8.21 (2H, d, *J* = 8.72 Hz, Ar‐H), 7.60 (1H, dd, *J* = 2.83, *J* = 11.29 Hz, Ar‐H), 7.44 (1H, dd, *J* = 6.30, *J* = 8.79 Hz, Ar‐H), 6.96 (1H, td, *J* = 2.91, *J* = 8.56 Hz, Ar‐H), 1.45 (9H, s, C‐(CH_3_)_3_); ^13^C NMR (101 MHz, DMSO‐*d*_6_) *δ* 164.8 (C), 154.2 (C), 150.1 (C), 141.1 (C), 133.3 (C), 131.6 (CH), 130.1 (CH), 129.9 (CH), 127.4 (C), 127.0 (C), 124.7 (CH), 124.4 (CH), 80.4 (C), 29.0 (CH_3_ x3); ^19^F NMR (376 MHz, DMSO‐*d*_6_) *δ* −73.6; *m/z* (ESI positive) 376.12 [M+H]^+^; Elemental analysis calculated (%):C, 57.60; H, 4.83; N, 11.19; found: C, 57.62; H, 4.81; N, 11.17.

#### 4.1.6 Synthesis of *Tert*‐Butyl [2‐(4‐Aminobenzamido)Phenyl]Carbamate **6** and *Tert*‐Butyl [2‐(4‐Aminobenzamido)‐5‐Fluorophenyl]Carbamate **7**

The appropriate nitro‐derivative *tert*‐butyl [2‐(4‐nitrobenzamido)phenyl]carbamate **4** or *tert*‐butyl [5‐fluoro‐2‐(4‐nitrobenzamido)phenyl]carbamate **5** (1.0 eq.) was dissolved in a mixture of H_2_O‐EtOH (2:1), followed by the addition of Fe (powder) (6.0 eq.) and CH_3_COOH (0.6 eq.) at room temperature. The reaction mixture was heated at 70°C and stirred for 4 h, cooled at room temperature, quenched with H_2_O, and extracted with EtOAc and dried over Na_2_SO_4_. The crude reaction was purified by flash chromatography (EtOAc/Hex 70:30) to afford *tert*‐butyl [2‐(4‐aminobenzamido)phenyl]carbamate **6** and *tert*‐butyl [2‐(4‐aminobenzamido)‐5‐fluorophenyl]carbamate **7**.

Following the above procedure, *tert*‐butyl [2‐(4‐nitrobenzamido)phenyl]carbamate **4** gave *tert*‐butyl [2‐(4‐aminobenzamido)phenyl]carbamate **6** as a white solid. Yield 41%. ^1^H NMR (400 MHz, DMSO‐*d*_6_) *δ* 9.47 (1H, s, exchange with D_2_O, –NH), 8.65 (1H, s, exchange with D_2_O, –NH), 7.68 (2H, d, *J* = 8.48 Hz, Ar‐H), 7.50 (2H, dd, *J* = 7.48, *J* = 22.59 Hz, Ar‐H), 7.14 (2H, m, Ar‐H), 6.60 (2H, d, *J* = 8.46 Hz, Ar‐H), 5.81 (2H, s, exchange with D_2_O, –NH_2_), 1.45 (9H, s, C‐(CH_3_)_3_); ^13^C NMR (101 MHz, DMSO‐*d*_6_) *δ* 166.5 (C), 161.7 (C), 159.5 (C), 153.9 (C), 153.3 (CH), 138.6 (C), 134.0 (C), 130.9 (CH), 128.8 (CH), 128.1 (CH), 120.5 (CH), 113.7 (CH), 81.4 (C), 29.3 (CH_3_ x3); *m/z* (ESI positive) 328.38 [M+H]^+^; Elemental analysis calculated (%):C, 66.04; H, 6.47; N, 12.84; found: C, 66.07; H, 6.50; N, 12.82.

Following the above procedure, *tert*‐butyl [5‐fluoro‐2‐(4‐nitrobenzamido)phenyl]carbamate **5** gave *tert*‐butyl [2‐(4‐aminobenzamido)‐5‐fluorophenyl]carbamate **7** as a yellow solid. Yield 32%. ^1^H NMR (400 MHz, DMSO‐*d*_6_) *δ* 9.44 (1H, s, exchange with D_2_O, –NH), 8.67 (1H, s, exchange with D_2_O, –NH), 7.68 (2H, d, *J* = 8.69 Hz, Ar‐H), 7.44 (2H, m, Ar‐H), 6.95 (1H, m, Ar‐H), 6.60 (2H, d, *J* = 8.69 Hz, Ar‐H), 5.80 (2H, s, exchange with D_2_O, –NH_2_), 1.46 (9H, s, C‐(CH_3_)_3_); ^13^C NMR (101 MHz, DMSO‐*d*_6_) *δ* 166.5 (C), 161.3 (C), 158.9 (C), 153.9 (C), 153.3 (CH), 134.6 (C), 134.5 (C), 130.3 (CH), 128.7 (CH), 128.6 (CH), 121.0 (CH), 113.5 (C), 81.0 (C), 28.9 (CH_3_ x3); ^19^F NMR (376 MHz, DMSO‐*d*_6_) *δ* –116.4; *m/z* (ESI positive) 346.37 [M+H]^+^; Elemental analysis calculated (%):C, 62.60; H, 5.84; N, 12.17; found: C, 62.61; H, 5.83; N, 12.15.

#### 4.1.7 Synthesis of Phenyl [4‐({2‐[(*Tert*‐Butoxycarbonyl)Amino]Phenyl}Carbamoyl)Phenyl]Carbamate **8** and Phenyl [4‐({2‐[(*Tert*‐Butoxycarbonyl)Amino]‐4‐Fluorophenyl}Carbamoyl)Phenyl]Carbamate **9**

The appropriate amino‐derivative *tert*‐butyl [2‐(4‐aminobenzamido)phenyl]carbamate **6** or *tert*‐butyl [2‐(4‐aminobenzamido)‐5‐fluorophenyl]carbamate **7** (1.0 eq.) was dissolved in THF, followed by the addition of DIPEA (1.5 eq.). Phenyl chloroformate (1.2 eq.) was added dropwise at 0°C, and the reaction mixture was stirred at room temperature for 4 h, concentrated under vacuum, quenched with H_2_O, and extracted with EtOAc and dried over Na_2_SO_4_. The crude reaction was purified by flash chromatography (EtOAc/Hex 50:50) to afford phenyl [4‐({2‐[(*tert*‐butoxycarbonyl)amino]phenyl}carbamoyl)phenyl]carbamate **8** and [4‐({2‐[(tert‐butoxycarbonyl)amino]‐4‐fluorophenyl}carbamoyl)phenyl]carbamate **9**. Following the above procedure, *tert*‐butyl [2‐(4‐aminobenzamido)phenyl]carbamate **6** gave phenyl [4‐({2‐[(*tert*‐butoxycarbonyl)amino]phenyl}carbamoyl)phenyl]carbamate **8** as a white solid. Yield 74%. ^1^H NMR (400 MHz, DMSO‐*d*_6_) *δ* 9.51 (1H, s, exchange with D_2_O, –NH), 9.38 (1H, s, exchange with D_2_O, –NH), 8.67 (1H, s, exchange with D_2_O, –NH), 7.71 (2H, d, *J* = 8.34 Hz, Ar‐H), 7.53 (2H, m, Ar‐H), 7.19 (3H, m, Ar‐H), 6.78 (2H, d, *J* = 7.72 Hz, Ar‐H), 6.65 (2H, d, *J* = 8.15 Hz, Ar‐H), 5.84 (2H, s, Ar‐H), 1.49 (9H, s, C‐(CH_3_)_3_); ^13^C NMR (101 MHz, DMSO‐*d*_6_) δ 165.7 (C), 154.4 (C), 152.6 (C), 151.3 (C), 143.0 (CH), 132.6 (CH), 132.4 (C), 130.8 (CH), 130.4 (C), 129.6 (CH), 129.3 (CH), 127.0 (CH), 126.5 (CH), 126.4 (CH), 125.0 (CH), 124.8 (CH), 122.9 (CH), 118.6 (C), 116.1 (C), 80.6 (C), 28.9 (CH_3_ x3); *m/z* (ESI positive) 448.49 [M+H]^+^; Elemental analysis calculated (%): C, 67.10; H, 5.63; N, 9.39; found: C, 67.15; H, 5.60; N, 9.37.

Following the above procedure, *tert*‐butyl [2‐(4‐aminobenzamido)‐5‐fluorophenyl]carbamate **7** gave phenyl [4‐({2‐[(*tert*‐butoxycarbonyl)amino]‐4‐fluorophenyl}carbamoyl)phenyl]carbamate **9** as a yellow solid. Yield 80%. ^1^H NMR (400 MHz, DMSO‐*d*_6_) *δ* 10.57 (1H, s, exchange with D_2_O, –NH), 9.71 (1H, s, exchange with D_2_O, –NH), 8.75 (1H, s, exchange with D_2_O, –NH), 7.96 (2H, d, *J* = 8.76 Hz, Ar‐H), 7.65 (2H, d, *J* = 8.80 Hz, Ar‐H), 7.52 (1H, dd, *J* = 2.95, *J* = 11.14 Hz, Ar‐H), 7.45 (3H, m, Ar‐H), 7.28 (3H, m, Ar‐H), 6.97 (1H, m, Ar‐H), 1.45 (9H, s, C‐(CH_3_)_3_); ^13^C NMR (101 MHz, DMSO‐*d*_6_) *δ* 166.1 (C), 159.3 (C), 154.0 (C), 152.6 (C), 151.3 (C), 142.9 (C), 130.4 (CH), 130.0 (CH), 129.3 (C), 126.6 (CH), 126.0 (CH), 122.9 (CH), 118.6 (C), 111.1 (C), 110.9 (CH), 110.1 (CH), 109.9 (CH), 81.0 (C), 29.0 (CH_3_ x3); ^19^F NMR (376 MHz, DMSO‐*d*_6_) *δ* –116.4; *m/z* (ESI positive) 466.48 [M+H]^+^; Elemental analysis calculated (%): C, 64.51; H, 5.20; N, 9.03; found: C, 64.53; H, 5.17; N, 9.01.

#### 4.1.8 Synthesis of *Tert*‐Butyl [2‐(4‐{3‐[2‐(1*H*‐Imidazol‐4‐yl)Ethyl]ureido}Benzamido)Phenyl]Carbamate **10** and *Tert*‐Butyl [2‐(4‐{3‐[2‐(1*H*‐Imidazol‐4‐yl)Ethyl]Ureido}Bbenzamido)‐5‐Fluorophenyl]Carbamate **11**

The appropriate derivative phenyl [4‐({2‐[(*tert*‐butoxycarbonyl)amino]phenyl}carbamoyl)phenyl]carbamate **8** or [4‐({2‐[(*tert*‐butoxycarbonyl)amino]‐4‐fluorophenyl}carbamoyl)phenyl]carbamate **9** (1.0 eq.) was dissolved in dry acetonitrile (5 mL) and 2‐(1*H*‐imidazol‐4‐yl)ethan‐1‐amine dihydrochloride was added (1.0 eq.). After Et_3_N addition (2 eq.), the reaction mixture was stirred at reflux for 18 h. After cooling the mixture to r.t., H_2_O was added, and the aqueous layer was extracted with EtOAc. The combined organic layers were dried over Na_2_SO_4_. The crude reaction was purified by flash chromatography (MeOH/DCM 5:95) to afford *tert*‐butyl [2‐(4‐{3‐[2‐(1*H*‐imidazol‐4‐yl)ethyl]ureido}benzamido)phenyl]carbamate **10** and *tert*‐butyl [2‐(4‐{3‐[2‐(1*H*‐imidazol‐4‐yl)ethyl]ureido}benzamido)‐5‐fluorophenyl]carbamate **11.**

Following the above procedure, phenyl [4‐({2‐[(*tert*‐butoxycarbonyl)amino]phenyl}carbamoyl)phenyl]carbamate **8** gave *tert*‐butyl [2‐(4‐{3‐[2‐(1*H*‐imidazol‐4‐yl)ethyl]ureido}benzamido)phenyl]carbamate **10** as a white solid. Yield 65%. ^1^H NMR (400 MHz, DMSO‐*d*_6_) *δ* 9.71 (1H, s, exchange with D_2_O, –NH), 9.14 (1H, s, exchange with D_2_O, –NH), 8.67 (1H, s, exchange with D_2_O, –NH), 7.85 (3H, m, Ar‐H), 7.52 (4H, ap t, *J* = 9.68 Hz, Ar‐H), 7.16 (2H, m, Ar‐H), 6.98 (1H, s), 6.50 (1H, m, exchange with D_2_O, –NH), 3.06 (2H, ap q, *J* = 7.31 Hz, CH_2_‐CH_2_), 2.71 (2H, t, *J* = 6.81 Hz, CH_2_‐CH_2_); 1.45 (9H, s, C‐(CH_3_)_3_); ^13^C NMR (101 MHz, DMSO‐*d*_6_) *δ* 165.8 (C), 155.8 (C), 154.4 (C), 145.0 (C), 135.4 (CH), 134.8 (C), 132.4 (C), 131.0 (C), 129.5 (CH), 127.0 (C), 126.8 (CH), 126.2 (CH), 125.0 (CH), 124.8 (CH), 117.5 (CH), 117.4 (CH), 80.5 (C), 46.4 (CH_2_), 29.0 c, 9.4 (CH_2_); *m/z* (ESI positive) 465.43 [M+H]^+^; Elemental analysis calculated (%): C, 62.06; H, 6.08; N, 18.09; found: C, 62.04; H, 6.07; N, 18.05.

Following the above procedure, phenyl [4‐({2‐[(*tert*‐butoxycarbonyl)amino]‐4‐fluorophenyl}carbamoyl)phenyl]carbamate **9** gave *tert*‐butyl [2‐(4‐{3‐[2‐(1*H*‐imidazol‐4‐yl)ethyl]ureido}benzamido)‐5‐fluorophenyl]carbamate **11** as a white solid. Yield 44%. ^1^H NMR (400 MHz, DMSO‐*d*_6_) δ 9.66 (1H, s, exchange with D_2_O, –NH), 9.05 (1H, m, exchange with D_2_O, –NH), 8.73 (1H, s, exchange with D_2_O, –NH), 7.86 (2H, d, *J* = 8.78 Hz, Ar‐H), 7.75 (1H, s, Ar‐H), 7.53 (2H, d, *J* = 8.81 Hz, Ar‐H), 7.47 (2H, m, Ar‐H), 6.96 (1H, m), 6.93 (1H, s, Ar‐H), 6.42 (1H, t, *J* = 5.30 Hz, exchange with D_2_O, –NH), 3.06 (2H, q, *J* = 7.27 Hz, CH_2_‐CH_2_), 2.70 (2H, t, *J* = 6.92 Hz, CH_2_‐CH_2_), 1.45 (9H, s, C‐(CH_3_)_3_); ^13^C NMR (101 MHz, DMSO‐*d*_6_) *δ* 166.7 (C), 155.5 (C), 144.8 (C), 134.3 (C), 132.6 (C), 129.6 (C), 129.0 (C), 129.9 (CH), 127.0 (C), 120.2 (CH), 117.2 (CH), 117.4 (CH), 103.5 (C), 102.9 (CH), 102.6 (CH), 102.3 (CH), 80.5 (C), 38.9 (CH_2_), 29.1 (CH_3_ x3), 26.6 (CH_2_); ^19^F NMR (376 MHz, DMSO‐*d*_6_) δ −115.9; *m/z* (ESI positive) 483.52 [M+H]^+^; Elemental analysis calculated (%): C, 59.74; H, 5.64; N, 17.42; found: C, 59.77; H, 5.63; N, 17.40.

#### 4.1.9 Synthesis of 2‐(4‐{3‐[2‐(1*H*‐Imidazol‐4‐yl)Ethyl]Ureido}Benzamido)Benzenaminium 2,2,2‐Trifluoroacetate **12** and 2‐(4‐{3‐[2‐(1*H*‐Imidazol‐4‐yl)Ethyl]Ureido}Benzamido)‐5‐Fluorobenzenaminium 2,2,2‐Trifluoroacetate **13**

The appropriate *tert*‐butyl [2‐(4‐{3‐[2‐(1*H*‐imidazol‐4‐yl)ethyl]ureido}benzamido)phenyl]carbamate **10** or *tert*‐butyl [2‐(4‐{3‐[2‐(1*H*‐imidazol‐4‐yl)ethyl]ureido}benzamido)‐5‐fluorophenyl]carbamate **11** (1.0 eq.) was dissolved in DCM (2 mL) at 0°C. Then, CF_3_COOH (5.0 eq.) was added. The temperature was raised up to r.t. and the reaction mixture was stirred for 3 h. The mixture was concentrated under vacuum, washed with Et_2_O, and dried on air. The product was pure enough to be used as it was.

Following the above procedure, *tert*‐butyl [2‐(4‐{3‐[2‐(1*H*‐imidazol‐4‐yl)ethyl]ureido}benzamido)phenyl]carbamate **10** gave 2‐(4‐{3‐[2‐(1*H*‐imidazol‐4‐yl)ethyl]ureido}benzamido)benzenaminium 2,2,2‐trifluoroacetate **12** as a white solid. Yield 78%. ^1^H NMR (400 MHz, DMSO‐*d*_6_) *δ* 9.51 (1H, s, exchange with D_2_O, –NH), 9.01 (2H, d, *J* = 14.45 Hz, Ar‐H), 7.88 (2H, d, *J* = 8.53 Hz, Ar‐H), 7.51 (1H, s, exchange with D_2_O, –NH), 7.49 (2H, s, Ar‐H), 7.16 (1H, d, *J* = 7.95 Hz, Ar‐H), 6.97 (1H, t, *J* = 7.57 Hz, exchange with D_2_O, –NH), 6.79 (1H, d, *J* = 7.89 Hz, Ar‐H), 6.62 (2H, t, *J* = 7.38 Hz, Ar‐H), 3.43 (4H, m, exchange with D_2_O, –NH_*2*_, CH_2_–CH_2_), 2.84 (2H, t, *J* = 6.57 Hz, CH_2_–CH_2_); ^13^C NMR (101 MHz, DMSO‐*d*_6_) *δ* 165.7 (C), 155.9 (C), 144.4 (C), 143.6 (C), 134.7 (C), 132.1 (C), 129.6 (CH), 127.6 (CH), 127.5 (CH), 127.1 (CH), 124.8 (CH), 117.5 (CH), 117.4 (CH), 117.3 (CH), 117.1 (C), 38.9 (CH_2_), 26.1 (CH_2_); ^19^F NMR (376 MHz, DMSO‐*d*_6_) *δ* –73.56; *m/z* (ESI positive) 479.43 [M+H]^+^; Elemental analysis calculated (%): C, 52.72; H, 4.42; N, 17.57; found: C, 52.70; H, 4.40; N, 17.56.

Following the above procedure, *tert*‐butyl [2‐(4‐{3‐[2‐(1*H*‐imidazol‐4‐yl)ethyl]ureido}benzamido)‐5‐fluorophenyl]carbamate **11** gave 2‐(4‐{3‐[2‐(1*H*‐imidazol‐4‐yl)ethyl]ureido}benzamido)‐5‐fluorobenzenaminium 2,2,2‐trifluoroacetate **13** as a white solid. Yield 69%. ^1^H NMR (400 MHz, DMSO‐*d*_6_) *δ* 9.41 (1H, s, exchange with D_2_O, –NH), 9.00 (1H, s, exchange with D_2_O, –NH), 8.95 (1H, s, exchange with D_2_O, –NH), 7.87 (2H, d, *J* = 8.58 Hz, Ar‐H), 7.48 (3H, m, Ar‐H), 7.09 (1H, dd, *J* = 6.53, *J* = 8.41 Hz, Ar‐H), 6.54 (2H, dd, *J* = 2.78, *J* = 11.16 Hz, Ar‐H), 6.35 (1H, td, *J* = 2.79, *J* = 8.51 Hz, Ar‐H), 5.17 (1H, br s, exchange with D_2_O, –NH_*2*_), 2.84 (2H, t, *J* = 6.64 Hz, CH_2_–CH_2_), 1.18 (2H, t, *J* = 7.28 Hz, CH_2_‐CH_2_); ^13^C NMR (101 MHz, DMSO‐*d*_6_) *δ* 166.1 (C), 156.0 (C), 144.5 (C), 134.8 (C), 132.1 (C), 129.7 (C), 129.5 (CH), 129.4 (CH), 127.5 (C), 120.6 (C), 117.4 (CH), 117.2 (CH), 103.1 (CH), 102.9 (CH), 102.6 (CH), 102.3 (CH), 38.9 (CH_2_), 26.1 (CH_2_); ^19^F NMR (376 MHz, DMSO‐*d*_6_) *δ* –73.56; *m/z* (ESI positive) 497.42 [M+H]^+^; Elemental analysis calculated (%): C, 50.81; H, 4.06; N, 16.93; found: C, 50.79; H, 4.05; N, 16.90.

#### 4.1.10 Synthesis of 4‐{3‐[2‐(1*H*‐Imidazol‐4‐yl)Ethyl]Ureido}‐*N*‐(2‐Amino‐4‐Fluorophenyl)Benzamide Diperchlorate **14**

2‐(4‐{3‐[2‐(1*H*‐Imidazol‐4‐yl)ethyl]ureido}benzamido)‐5‐fluorobenzenaminium 2,2,2‐trifluoroacetate **13** (1.0 eq.) was dissolved in H_2_O and sodium perchloride (5.0 eq.) was added, and the temperature raised up to 55°C for 1 h. After cooled to r.t., the reaction mixture was concentrated under vacuum, washed with Et_2_O, and dried on air. The product was pure enough to be used as it was. White solid. Yield 70%. ^1^H NMR (400 MHz, DMSO‐*d*_6_) *δ* 14.07 (1H, brs, exchange with D_2_O, –NH), 9.41 (1H, s, exchange with D_2_O, –NH), 8.97 (1H, s, Ar‐H), 8.80 (1H, s, exchange with D_2_O, –NH), 7.87 (2H, d, *J* = 8.78 Hz, Ar‐H), 7.48 (3H, m, Ar‐H), 7.09 (1H, t, *J* = 6.47 Hz, exchange with D_2_O, –NH), 6.54 (1H, dd, *J* = 2.48 Hz, *J* = 11.35 Hz, Ar‐H), 6.37 (2H, m, Ar‐H), 5.17 (2H, brs, exchange with D_2_O, –NH_2_), 3.42 (2H, dd, *J* = 6.58 Hz, *J* = 12.51 Hz, CH_2_‐CH_2_), 2.84 (2H, t, *J* = 6.50 Hz, CH_2_‐CH_2_); ^13^C NMR (101 MHz, DMSO‐*d*_6_) *δ* 166.0 (C), 161.8 (d, ^1^ *J*_C‐F_ = 238 Hz, C), 155.8 (C), 146.3 (d, ^3^ *J*_C‐F_ = 11 Hz, C), 144.3 (C), 134.8 (C), 132.1 (C), 129.7 (CH), 129.3 (d, ^3^ *J*_C‐F_ = 11 Hz, CH), 127.6 (CH), 120.5 (d, ^4^ *J*_C‐F_ = 2 Hz, C), 117.5 (CH), 117.1 (CH), 103.0 (d, ^2^ *J*_C‐F_ = 22 Hz, CH), 102.4 (d, ^2^ *J*_C‐F_ = 22 Hz, CH), 38.9 (CH_2_), 26.1 (CH_2_); ^19^F NMR (376 MHz, DMSO‐*d*_6_) δ ‐116.70; *m/z* (ESI positive) 584.31 [M+H]^+^; Elemental analysis calculated (%): C, 39.12; H, 3.63; N, 14.41; found: C, 39.15; H, 3.65; N, 14.39.

#### 4.1.11 Synthesis of [4‐(Methoxycarbonyl)Phenyl]Glycine **15**

Methyl 4‐[(2‐ethoxy‐2‐oxoethyl)amino]benzoate (1.0 eq.) was dissolved in EtOH and 5% aqueous NaOH (1.1 eq.) was added. The reaction mixture was vigorously stirred for 5 min, then quenched with 1 N HCl until pH = 3, extracted with EtOAc, and dried over Na_2_SO_4_. The product was pure enough to be used as it was. Yellow solid. Yield 51%. ^1^H NMR (400 MHz, DMSO‐*d*_6_) *δ* 12.67 (1H, brs, exchange with D_2_O, –COO*H*), 7.79 (2H, d, *J* = 8.88 Hz, Ar‐H), 6.76 (1H, brs, exchange with D_2_O, –NH), 6.59 (2H, d, *J* = 8.85 Hz, Ar‐H), 3.89 (2H, m, –CH_2_), 3.74 (3H, s, –CH_3_); ^13^C NMR (101 MHz, DMSO‐*d*_6_) *δ* 172.9 (C), 167.2 (C), 153.3 (C), 131.8 (C), 117.4 (CH), 112.2 (CH_3_), 52.1 (CH), 45.0 (CH_2_); *m/z* (ESI positive) 210.20 [M+H]^+^; Elemental analysis calculated (%): C, 57.41; H, 5.30; N, 6.70; found: C, 57.39; H, 5.27; N, 6.71.

#### 4.1.12 Synthesis of Methyl 4‐({2‐[(2,5‐Dioxopyrrolidin‐1‐yl)Oxy]‐2‐Oxoethyl}Amino)Benzoate **16**

[4‐(Methoxycarbonyl)phenyl]glycine **15** (1.0 eq.) was dissolved in dimethylacetamide (2 mL), then *N*‐hydroxysuccinimide (1.5 eq.) and EDCI (1.5 eq.) were added at 0°C. The temperature was raised up to r.t. and the reaction mixture was stirred for 2 h, quenched with slush, extracted with DCM, and dried over Na_2_SO_4_. The product was pure enough to be used as it was. White solid. Yield 72%. ^1^H NMR (400 MHz, DMSO‐*d*_6_) *δ* 7.72 (2H, d, *J* = 8.83 Hz, Ar‐H), 7.10 (1H, t, *J* = 6.30 Hz, exchange with D_2_O, –NH), 6.66 (2H, d, *J* = 8.82 Hz, Ar‐H), 4.48 (2H, d, *J* = 6.42 Hz, –CH_2_), 3.75 (3H, s, –CH_3_), 2.81 (4H, m, CH_2_‐CH_2_ x2); ^13^C NMR (101 MHz, DMSO‐*d*_6_) δ 171.0 (C), 168.2 (C), 167.1 (C), 152.6 (C), 131.7 (CH), 118.4 (CH), 112.5 (CH_3_), 52.2 (CH_2_), 42.8 (CH_2_), 26.4 (CH_2_); *m/z* (ESI positive) 307.27 [M+H]^+^; Elemental analysis calculated (%): C, 54.90; H, 4.61; N, 9.15; found: C, 54.87; H, 4.59; N, 9.18.

#### 4.1.13 Synthesis of Methyl 4‐{[2‐Oxo‐2‐(Phenethylamino)Ethyl]Amino}Benzoate **17**

Under an inert atmosphere (N_2_), 4‐({2‐[(2,5‐dioxocyclopentyl)oxy]‐2‐oxoethyl}amino)benzoate **16** (1.0 eq.) and 2‐phenylethan‐1‐amine (1.1 eq.) were dissolved in dry dimethylacetamide (2 mL). Then Et_3_N (2.0 eq.) was added, and the reaction mixture was stirred overnight at r.t., quenched with slush, extracted with EtOAc, and dried over Na_2_SO_4_. The crude product was purified by flash chromatography (EtOAc/Hex 50:50) to afford the pure compound. Yellow solid. Yield 47%. ^1^H NMR (400 MHz, DMSO‐*d*_6_) *δ* 7.99 (1H, t, *J* = 5.55 Hz, exchange with D_2_O, –NH), 7.69 (2H, d, *J* = 8.79 Hz, Ar‐H), 7.26 (2H, t, *J* = 7.21 Hz, Ar‐H), 7.17 (3H, ap t, *J* = 6.27 Hz, Ar‐H), 6.77 (1H, t, *J* = 5.90 Hz, exchange with D_2_O, –NH), 6.54 (2H, d, *J* = 8.79 Hz, Ar‐H), 3.75 (3H, s, –CH_3_), 3.69 (2H, d, *J* = 6.01 Hz, –CH_2_), 3.34 (2H, m, CH_2_–CH_2_), 2.70 (2H, t, *J* = 7.31 Hz, CH_2_–CH_2_); ^13^C NMR (101 MHz, DMSO‐*d*_6_) *δ* 170.2 (C), 167.2 (C), 153.4 (C), 140.2 (C), 131.7 (C), 129.5 (CH), 129.2 (CH), 127.0 (CH), 117.5 (CH), 112.3 (CH), 52.2 (CH_3_), 47.0 (CH_2_), 41.0 (CH_2_), 36.0 (CH_2_); *m/z* (ESI positive) 313.37 [M+H]^+^; Elemental analysis calculated (%): C, 69.21; H, 6.45; N, 8.97; found: C, 69.19; H, 6.43; N, 8.98.

#### 4.1.14 Synthesis of 4‐{[2‐Oxo‐2‐(Phenethylamino)Ethyl]Amino}Benzoic Acid **18**

Methyl 4‐{[2‐oxo‐2‐(phenethylamino)ethyl]amino}benzoate **17** (1.0 eq.) was dissolved in THF/MeOH/H_2_O (2:2:1) and LiOH (6.0 eq.). The mixture was stirred at room temperature overnight until the consumption of the starting material. The reaction was quenched with 1 N aqueous HCl, extracted with EtOAc, and dried over Na_2_SO_4_. The crude material was purified by flash column chromatography (MeOH/DCM: 5:95) to yield the pure compound. White solid. Yield 89%. ^1^H NMR (400 MHz, DMSO‐*d*_6_) *δ* 12.06 (1H, brs, exchange with D_2_O, –COO*H*), 7.99 (1H, t, *J* = 5.95 Hz, exchange with D_2_O, –NH), 7.67 (2H, d, *J* = 8.83 Hz, Ar‐H), 7.26 (2H, t, *J* = 7.13 Hz, Ar‐H), 7.17 (3H, m, Ar‐H), 6.69 (1H, t, *J* = 5.73 Hz, exchange with D_2_O, –NH), 6.52 (2H, d, *J* = 8.88 Hz, Ar‐H), 3.68 (2H, d, *J* = 5.99 Hz, –CH_2_), 3.30 (2H, m, CH_2_–CH_2_), 2.70 (2H, t, *J* = 7.25 Hz, CH_2_‐CH_2_); ^13^C NMR (101 MHz, DMSO‐*d*_6_) *δ* 170.3 (C), 168.3 (C), 153.1 (C), 140.2 (C), 131.9 (CH), 129.5 (C), 129.2 (CH), 126.9 (CH), 118.7 (CH), 112.2 (CH), 47.1 (CH_2_), 41.0 (CH_2_), 36.0 (CH_2_); *m/z* (ESI positive) 299.34 [M+H]^+^; Elemental analysis calculated (%): C, 68.44; H, 6.08; N, 9.39; found: C, 68.41; H, 6.06; N, 9.37.

#### 4.1.15 Synthesis of *N*‐(2‐Aminophenyl)‐4‐{[2‐Oxo‐2‐(Phenethylamino)Ethyl]Amino}Benzamide **19**

Under an inert atmosphere (N_2_), 4‐{[2‐oxo‐2‐(phenethylamino)ethyl]amino}benzoic acid **18** (1.0 eq.) and 1‐hydroxy‐7‐azabenzotriazole (HOAt) (1.5 eq.) were dissolved in dry dimethylacetamide (2 mL) and stirred for 15 min. Then *o*‐phenylenediamine (1.1 eq.) and EDCI (2.0 eq.) were added at 0°C, followed by Et_3_N (4.0 eq.) addition. The reaction mixture was stirred at r.t. for 6 h, quenched with slush, and the precipitate solid was collected via filtration, washed with H_2_O, and dried on air. The product was pure enough to be used as it was. Yellow solid. Yield 61%. ^1^H NMR (400 MHz, DMSO‐*d*_6_) *δ* 9.35 (1H, s, exchange with D_2_O, –NH), 8.03 (1H, t, exchange with D_2_O, –NH), 7.83 (2H, d, *J* = 8.38 Hz, Ar‐H), 7.32 (2H, t, *J* = 7.19 Hz, Ar‐H), 7.22 (4H, dd, *J* = 10.33 Hz, *J* = 18.15 Hz, Ar‐H), 6.98 (1H, t, *J* = 7.47 Hz, exchange with D_2_O, –NH), 6.81 (1H, d, *J* = 7.91 Hz, Ar‐H), 6.63 (3H, m, Ar‐H), 6.56 (1H, t, *J* = 5.74 Hz, Ar‐H), 4.86 (2H, s, exchange with D_2_O, –NH_*2*_), 3.74 (2H, d, *J* = 5.74 Hz, –CH_2_), 3.38 (2H, m, CH_2_–CH_2_), 2.76 (2H, t, *J* = 7.22 Hz, CH_2_–CH_2_); ^13^C NMR (101 MHz, DMSO‐*d*_6_) *δ* 170.4 (C), 165.9 (C), 153.3 (C), 152.0 (C), 143.8 (CH), 140.2 (C), 131.7 (C), 130.1 (CH), 129.5 (CH), 129.2 (CH), 127.3 (CH), 126.9 (CH), 125.1 (CH), 122.7 (CH), 117.1 (CH), 112.1 (C), 52.1 (CH_2_), 47.2 (CH_2_), 36.1 (CH_2_); *m/z* (ESI positive) 389.47 [M+H]^+^; Elemental analysis calculated (%):C, 71.11; H, 6.23; N, 14.42; found: C, 71.09; H, 6.25; N, 14.43.

### 4.2 In Vitro Measurement of Enzymatic Activity

#### 4.2.1 CA In Vitro Assessment

An Applied Photophysics stopped‐flow instrument was used to assay the CA‐catalyzed CO_2_ hydration activity [30]. Phenol red (at a concentration of 0.2 mM) was used as an indicator, working at the absorbance maximum of 557 nm, with 20 mM 4‐(2‐hydroxyethyl)‐1‐piperazineethanesulfonic acid (HEPES) (pH 7.4) as a buffer, and 20 mM Na_2_SO_4_ (to maintain constant ionic strength), following the initial rates of the CA‐catalyzed CO_2_ hydration reaction for a period of 10–100 s. The CO_2_ concentrations ranged from 1.7 to 17 mM for the determination of the kinetic parameters and inhibition constants. Enzyme concentrations ranged between 5 and 12 nM. For each inhibitor, at least six traces of the initial 5%–10% of the reaction were used to determine the initial velocity. The uncatalyzed rates were determined in the same manner and subtracted from the total observed rates. Stock solutions of the inhibitor (0.1 mM) were prepared in distilled–deionized water and dilutions up to 0.01 nM were done thereafter with the assay buffer. Inhibitor and enzyme solutions were preincubated together for 15 min at r.t. before the assay, to allow for the formation of the E–I complex. The inhibition constants were obtained by nonlinear least‐squares methods using PRISM 3 and the Cheng‐Prusoff equation [41] and represent the mean from at least three different determinations [34, 42]. Apart from commercial hCAs I and II, all CA isoforms were recombinant proteins obtained in house, as reported earlier [43, 44].

#### 4.2.2 HDAC In Vitro Assessment

HDAC‐inhibitory activity was evaluated using a fluorometric activity assay kit purchased from Enzo Life Sciences “Fluor de Lys‐Green HDAC assay kit, BML‐AK530” (Farmingdale, NY). HeLa nuclear extract, rich in HDAC activity, is included with the kit. Trichostatin A solution was used as a positive control and model inhibitor. Plates were read with excitation at 380 nm and emission at 440 nm on a TECAN Spark multimode microplate reader. Histone deacetylase activity was measured according to the manufacturer's instructions [45].

### 4.3 Ex Vivo Measurement of Enzymatic Activity in Brain Samples

#### 4.3.1 Animals

Adult male C57Bl6 (20–25 g, 8–9 weeks old, 25–30 g) were used in this study. They were housed at the animal facility of the Centro di Servizi per la Stabulazione di Animali da Laboratorio (CeSAL) of the University of Florence, in humidity and temperature‐controlled rooms (22 ± 2°C) with free access to food (4RF21; Mucedola s.r.l., Italy) and water, and kept on a 12‐h light/dark cycle (lights start at 8:00 a.m.). Animals were handled for 4 days before the beginning of the experiments to acclimatize to human contact. Housing and experimental procedures were conducted in accordance with the Council Directive of the European Community (2010/63/EU) and the Italian Decreto Legislativo 26 (13/03/2014) regarding the protection of animals used for scientific purposes, approved by the Animal Care Committee of the University of Florence and Italian Ministry of Health (797‐2021‐PR prot. 17E9C.233) and supervised by a veterinarian. Every effort was made to minimize animal suffering and to reduce the number of animals used, complying with the 3 R principle. Male mice were used to reduce within‐group variability due to hormonal fluctuations during oestrous cycle in female mice. All the experiments were performed between 9:00 a.m. and 2:00 p.m.

#### 4.3.2 Stereotaxic Surgery and Infusion Procedure

These procedures were performed following the protocols previously described [39, 46, 47]. In brief, animals were anesthetized using a mixture of ketamine (15 mg/kg) and xylazine (2.5 mg/kg) and placed in a stereotaxic frame equipped with a mouse adapter (Stoelting, Chicago, USA). A stainless steel cannula (7 mm in length, outer diameter 0.5 mm, and inner diameter 0.25 mm) was then implanted in the lateral ventricle and fixed to the skull using dental cement. The following coordinates were used according to the mouse brain atlas [48] antero‐posterior (AP) –0.3 mm; lateral (L) + 1 mm; dorsoventral (DV) –1 mm. After the surgery, animals were left to recover for 7 days before the start of the experiments.

In the infusion days, a stainless‐steel injection micro‐needle (outer diameter 0.25 mm) connected through a polyethylene catheter to a 1000 µL Hamilton precision syringe was inserted into the cannulae previously implanted and lowered into the lateral cerebral ventricle (DV 2.4 mm). Then, 5 µL of a solution containing the testing compounds or vehicle were delivered using an infusion pump at 1 µL/min flow. The needle was left in place for one additional minute after each infusion to avoid efflux. Thirty minutes after drug injections, the animals were euthanized, their brain immediately removed, the cortices and hippocampi were rapidly dissected on ice, weighted, and stored at –80°C until use.

The solutions containing compounds **12**, **14**, and **19** (synthesized as described above) and d‐Phe (purchased from Sigma‐Aldrich) were dissolved in physiological saline containing 1% of DMSO (v/v) at a final concentration of 50 nmol/µL and freshly prepared during each infusion day. Animals pertaining to the control group received infusions of vehicle (physiological saline containing 1% DMSO (v/v)).

#### 4.3.3 CA Activity

Brain tissues were homogenized in 1.0 mL of ice‐cold 20 mM Hepes buffer (pH 7.5). After centrifugation at 20,000*g* for 30 min, an Applied Photophysics stopped‐flow instrument was used for assaying the CA‐catalyzed CO_2_ hydration activity from the samples as previously described [23, 34]. Briefly, phenol red (at a concentration of 0.2 mM) was used as an indicator working at the absorbance maximum of 557 nm, with 20 mM Hepes buffer (pH 7.5) and 20 mM NaClO_4_ for maintaining constant ionic strength, following the initial rates of the CA‐catalyzed CO_2_ hydration reaction for a period of 10–100 s, at 20°C. The CO_2_ concentrations ranged from 1.7 to 17 mM for the determination of the kinetic parameters and activation constants. The mean CA activity determined in the brain samples from the control group of mice treated with vehicle was set as 100% and all the individual calculated values were then expressed as a percentage of controls.

#### 4.3.4 HDAC Activity

The residual enzymatic activity in brain tissues was measured using Fluor de Lys‐Green HDAC fluorometric activity assay kit purchased from Enzo Life Sciences (Farmingdale, NY). For the evaluation of the absorbance, disposable transparent cuvettes (12.5 × 12.5 × 45 mm) from Sarstedt were used. Measurements were performed using a TECAN Spark multimode microplate reader, set to a wavelength of 280 nm. Once the absorbance was measured, the concentration of the enzyme HDAC contained in the brain homogenates was calculated according to Lambert‐Beer's law. For determination of HDAC residual activity, brain tissues were homogenized in 1 mL of ice‐cold 20 mM Hepes buffer (pH 7.5). After centrifugation at 20,000*g* for 30 min, a fluorescent method assay was performed in a reaction volume of 100 μL in 96‐well microplates (PerkinElmer). After normalization, the final concentration of all HDACs in the enzymatic assays was between 1 and 5 µM. A final substrate concentration of 2 mM (2x the desired final concentration) was obtained by dilution with HDAC assay buffer (20 mL; 50 mM TRIS/Cl, pH 8.0, 137 mM NaCl, 2.7 mM KCl, 1 mM MgCl_2_). Substrate was then added to all homogenates and left incubating for 15 min. The reaction was terminated and developed by the addition of Fluor de Lys Developer previously diluted in HDAC assay buffer. Fluorescence was measured at 380 nm and detected at 440 nm. Each test included a blank as a reference for minimal fluorescence, containing only buffer and substrate, thus lacking any protein component [49, 50]. The final data are expressed as the percentage of residual HDAC activity in treated homogenates compared to control ones, whose value is set to 100%.

### 4.4 Investigation of Procognitive Effects Using the Novel Object Recognition (NOR) Paradigm

Male C57Bl6 mice were submitted to the stereotaxic surgery to implant a guide cannula into the brain lateral ventricles as described above. One week after the surgery, their behavior was assessed in an open‐field arena (60 × 70 cm and 30 cm high) constructed in white acrylic Plexiglass and placed in a sound‐attenuated room. The detailed experimental procedure is already published [35, 51, 52]. The NOR protocol consists of three sessions: habituation, acquisition, and retention test separated by 24 h intertrial intervals. During habituation, the animals freely explored the empty arena for 10 min. Twenty‐four hours later, each animal was placed in the same position and facing the same direction into the test arena in the presence of two identical objects (gray plastic shapes such as cubes, cylinders, or pyramids 8 cm high) and left free to explore it for 5 min. Immediately after this session, 5 μL vehicle of the freshly prepared solutions containing the compounds **12**, **14**, **19**, and d‐Phe at 0.5, 5, and 50 nmol/μL final concentrations were infused directly into the lateral ventricles through the previously implanted cannula. The retention test session was performed 24 h after acquisition, during which, each mouse was again placed in the test arena for 5 min in the presence of one of the familiar object and a novel object. The position of the new objects (left/right) was randomized to prevent bias from order or place preference. Animal's behavior during all session was videotaped, and the time spent actively exploring each object was recorded by an experienced observer unaware of the experimental groups. Exploration was defined as sniffing or touching the stimulus object with the nose and/or forepaws. Sitting on or turning around the objects was not considered exploratory behavior. Each animal was subjected to the procedure separately, and care was taken to remove any olfactory/taste cues by cleaning carefully the arena and test objects between trials with a solution of EtOH (30% v/v). Mice were placed in their home cages between trials. The final data are expressed as the percentage of time exploring the familiar and new objects during the retention test. The raw exploration time data were employed also to calculate the DI, according to the following equation:

$$\mathrm{Discrimination}\;\mathrm{Index}\;\left(DI\right)=\frac{\mathrm{time}\;\mathrm{exploring}\;\mathrm{novel}\;\left(tN\right)-\mathrm{time}\;\mathrm{exploring}\;\mathrm{familiar}\;(tF)}{\mathrm{total}\;\mathrm{exploration}\;\mathrm{time}\;(tN+tF)}.$$

### 4.5 Statistical Analysis

Data from behavioral and neurochemical experiments are expressed as individual points for each animal (scattered plot). The bar graph represents means ± standard error of the mean (S.E.M). These data were analyzed using GraphPad Software (version 10.1). The percentage of time the animals spent exploring the different objects was analyzed using two‐way ANOVA. The DIs and the percentage of enzymatic activity measured Ex Vivo in brain homogenates were analyzed by one‐way ANOVA. The source of the detected significances was determined using Bonferroni's multiple comparison post‐hoc test. *p* values lower than 0.05 were considered statistically significant. The sample size of each experimental group is indicated in the respective figure legends.

## Ethics Statement

Housing and experimental procedures were conducted in accordance with the Council Directive of the European Community (2010/63/EU) and the Italian Decreto Legislativo 26 (13/03/2014) regarding the protection of animals used for scientific purposes, approved by the Animal Care Committee of the University of Florence and Italian Ministry of Health (797‐2021‐PR prot. 17E9C.233) and supervised by a veterinarian. Every effort was made to minimize animal suffering and to reduce the number of animals used, complying with the 3 R principle.

## Conflicts of Interest

The authors declare no conflicts of interest.

## Supporting information

InChI file.

SI.

## Data Availability

The study protocol and all data collected, including raw data and their analyses, will be made available upon request to the corresponding author.
