# Supplementary material for: Hitting Two Birds With One Stone: Dual Modulation of Brain Carbonic Anhydrases and Histone Deacetylases Boosts Memory Consolidation
Source: Arch Pharm (Weinheim). 2025 Jun 18;358(6):e70020. doi: 10.1002/ardp.70020 (PMC12174897; doi:10.1002/ardp.70020)
Supplement: Supplementary file 1 — InChI file. [file ARDP-358-e70020-s002.doc]

**Supplemental Material: Novel Compounds and Biological Screening Results**

**Title of Manuscript** HITTING TWO BIRDS WITH ONE STONE: DUAL MODULATION OF BRAIN CARBONIC ANHYDRASES AND HISTONE DEACETYLASES BOOSTS MEMORY CONSOLIDATION

**Authors** Alessia Costa^1,*^ Murat Bozdag^2,*^, Gioele Renzi^2,*^, Barbara Rani^1^, Maria Beatrice Passani^3^, Andrea Angeli^2^, Gustavo Provensi^1,#^, Fabrizio Carta^2,#^ and Claudiu T. Supuran^2^

**Affiliations**

^1^ - Department of NEUROFARBA, Section of Pharmacology and Toxicology, Laboratory of Ocular and Neuropsychopharmacology (Braeye Lab), University of Florence, Viale Pieraccini 6, 50139, Florence, Italy

^2^ - NEUROFARBA Department, Pharmaceutical and Nutraceutical Section, University of Florence, Via Ugo Schiff 6, 50019, Sesto Fiorentino, Florence, Italy

^3^ - Department of Health Sciences, Laboratory of Ocular and Neuropsychopharmacology (Braeye Lab), University of Florence, Viale Pieraccini 6, 50139, Florence, Italy

* these authors contributed equally for this work

# corresponding authors

fabrizio.carta@unifi.it, gustavo.provensi@unifi.it

| **Cmp.** | **InChI** | **Biological Activity** |
| --- | --- | --- |
| **12** | InChI=1S/C19H20N6O2.2C2HF3O2/c20-16-3-1-2-4-17(16)25-18(26)13-5-7-14(8-6-13)24-19(27)22-10-9-15-11-21-12-23-15;2*3-2(4,5)1(6)7/h1-8,11-12H,9-10,20H2,(H,21,23)(H,25,26)(H2,22,24,27);2*(H,6,7) | ^a^hCA I *K*_I_ = 75.4 µM  ^a^hCA II *K*_I_ = 66.8 µM  ^a^hCA IV *K*_I_ = 23.0 µM  ^a^hCA VA *K*_I_ = >10000 µM  ^a^hCA VII *K*_I_ = 32.0 µM  ^a^hCA IX *K*_I_ = 68.8 µM  ^b^HDAC EC_50_ = 2.8 µM |
| **14** | InChI=1S/C19H19FN6O2.2ClHO4/c20-13-3-6-17(16(21)9-13)26-18(27)12-1-4-14(5-2-12)25-19(28)23-8-7-15-10-22-11-24-15;2*2-1(3,4)5/h1-6,9-11H,7-8,21H2,(H,22,24)(H,26,27)(H2,23,25,28);2*1H | ^a^hCA I *K*_I_ = 53.8 µM  ^a^hCA II *K*_I_ = 49.5 µM  ^a^hCA IV *K*_I_ = 49.1 µM  ^a^hCA VA *K*_I_ = 37.9 µM  ^a^hCA VII *K*_I_ = 26.1 µM  ^a^hCA IX *K*_I_ = >10000 µM  ^b^HDAC EC_50_ = 5.3 µM |
| **19** | InChI=1S/C23H24N4O2/c24-20-8-4-5-9-21(20)27-23(29)18-10-12-19(13-11-18)26-16-22(28)25-15-14-17-6-2-1-3-7-17/h1-13,26H,14-16,24H2,(H,25,28)(H,27,29) | ^a^hCA I *K*_I_ = 10000 µM  ^a^hCA II *K*_I_ = 10000 µM  ^a^hCA IV *K*_I_ = 10000 µM  ^a^hCA VA *K*_I_ = 10000 µM  ^a^hCA VII *K*_I_ = 10000 µM  ^a^hCA IX *K*_I_ = 10000 µM  ^b^HDAC EC_50_ = 14.2 µM |

**^a^ Carbonic Anhydrase in vitro assessment**

An Applied Photophysics stopped-flow instrument was used to assay the CA-catalyzed CO2 hydration activity [1]. Phenol red (at a concentration of 0.2 mM) was used as an indicator, working at the absorbance maximum of 557 nm, with 20 mM 4-(2-hydroxyethyl)-1-piperazineethanesulfonic acid (HEPES) (pH 7.4) as a buffer, and 20 mM Na_2_SO_4_ (to maintain constant ionic strength), following the initial rates of the CA-catalyzed CO_2_ hydration reaction for a period of 10–100 s. The CO_2_ concentrations ranged from 1.7 to 17 mM for the determination of the kinetic parameters and inhibition constants. Enzyme concentrations ranged between 5 and 12 nM. For each inhibitor, at least six traces of the initial 5–10% of the reaction were used to determine the initial velocity. The uncatalyzed rates were determined in the same manner and subtracted from the total observed rates. Stock solutions of the inhibitor (0.1 mM) were prepared in distilled–deionized water and dilutions up to 0.01 nM were done thereafter with the assay buffer. Inhibitor and enzyme solutions were preincubated together for 15 min at r.t. prior to the assay, to allow for the formation of the E–I complex. The inhibition constants were obtained by nonlinear least-squares methods using PRISM 3 and the Cheng-Prusoff equation [2] and represent the mean from at least three different determinations [3, 4]. Apart from commercial hCAs I and II, all CA isoforms were recombinant proteins obtained in house, as reported earlier [5, 6].

*^b^ Histone Deacetylases in vitro assessment*

HDAC-inhibitory activity was evaluated using a fluorometric activity assay kit purchased from Enzo Life Sciences “Fluor de Lys®-Green HDAC assay kit, BML-AK530” (Farmingdale, NY). HeLa nuclear extract, rich in HDAC activity, is included with the kit. Trichostatin A solution was used as a positive control and model inhibitor. Plates were read with excitation at 380 nm and emission at 440 nm on a TECAN Spark® multimode microplate reader. Histone deacetylase activity was measured according to manufacturer's instructions [7].

**References**

1. Khalifah, R.G. The carbon dioxide hydration activity of carbonic anhydrase. I. Stop-flow kinetic studies on the native human isoenzymes B and C*.* *J Biol Chem*, **1971**. *246*, 2561-2573.

2. Cheng, Y.; Prusoff, W.H. Relationship between the inhibition constant (K1) and the concentration of inhibitor which causes 50 per cent inhibition (I50) of an enzymatic reaction*.* *Biochem Pharmacol*, **1973**, *22*, 3099-3108.

3. Provensi, G.; Costa, A.; Rani, B.; Becagli, M. V.; Vaiano, F.; Passani, M. B.; Tanini, D.; Capperucci, A.; Carradori, S.; Petzer, J. P.; Petzer, A.; Vullo, D.; Costantino, G.; Blandina, P.; Angeli, A.; Supuran, C. T. New β-arylchalcogeno amines with procognitive properties targeting Carbonic Anhydrases and Monoamine Oxidases*.* *Eur J Med Chem*, **2022**, *244*, 114828.

4. Provensi, G.; Nocentini, A.; Passani, M. B.; Blandina, P.; Supuran, C. T. Activation of carbonic anhydrase isoforms involved in modulation of emotional memory and cognitive disorders with histamine agonists, antagonists and derivatives*.* *J Enzyme Inhib Med Chem*, **2021**. *36*, 719-726.

5. Aslan, H.; Renzi, G.; Angeli, A.; D'Agostino, I.; Ronca, R.; Massardi, M. L.; Tavani, C.; Carradori, S.; Ferraroni, M.; Governa, P.; Manetti, F.; Carta, F.; Supuran, C. T. Benzenesulfonamide decorated dihydropyrimidin(thi)ones: carbonic anhydrase profiling and antiproliferative activity*.* *RSC Med Chem*, **2024**. *15*, 1929-1941.

6. Benito, G.; D'Agostino, I.; Carradori, S.; Fantacuzzi, M.; Agamennone, M.; Puca, V.; Grande, R.; Capasso, C.; Carta, F.; Supuran, C. T. Erlotinib-containing benzenesulfonamides as anti- Helicobacter pylori agents through carbonic anhydrase inhibition*.* *Future Med Chem*, **2023**. *15*, 1865-1883.

7. Shao, M.; He, L.; Zheng, L.; Huang, L.; Zhou, Y.; Wang, T.; Chen, Y.; Shen, M.; Wang, F.; Yang, Z.; Chen, L. Structure-based design, synthesis and in vitro antiproliferative effects studies of novel dual BRD4/HDAC inhibitors*.* *Bioorg Med Chem Lett*, **2017**. *27*, 4051-4055.
