# Supplementary material for: Hitting Two Birds With One Stone: Dual Modulation of Brain Carbonic Anhydrases and Histone Deacetylases Boosts Memory Consolidation
Source: Arch Pharm (Weinheim). 2025 Jun 18;358(6):e70020. doi: 10.1002/ardp.70020 (PMC12174897; doi:10.1002/ardp.70020)
Supplement: Supplementary file 2 — SI. [file ARDP-358-e70020-s001.docx]

Supplementary Material for

**HITTING TWO BIRDS WITH ONE STONE: DUAL MODULATION OF BRAIN CARBONIC ANHYDRASES AND HISTONE DEACETYLASES BOOSTS RECOGNITION MEMORY CONSOLIDATION**

Alessia Costa^1,*^, Murat Bozdag^2*^, Gioele Renzi^2,*^, Barbara Rani^1^, Maria Beatrice Passani^3^, Gustavo Provensi^1,#^, Fabrizio Carta^2,#^ and Claudiu T. Supuran^2^

^1^ - Department of NEUROFARBA, Section of Pharmacology and Toxicology, Laboratory of Ocular and Neuropsychopharmacology (Braeye Lab), University of Florence, Viale Pieraccini 6, 50139, Florence, Italy

^2^ - NEUROFARBA Department, Pharmaceutical and Nutraceutical Section, University of Florence, Via Ugo Schiff 6, 50019, Sesto Fiorentino, Florence, Italy

^3^ - Department of Health Sciences, Laboratory of Ocular and Neuropsychopharmacology (Braeye Lab), University of Florence, Viale Pieraccini 6, 50139, Florence, Italy

* these authors contributed equally for this work

# corresponding authors: (G.P) [gustavo.provensi@unifi.it](mailto:gustavo.provensi@unifi.it) and (F.C.) [fabrizio.carta@unifi.it](mailto:fabrizio.carta@unifi.it),

Index

EC_50_ curve for compounds **12, 14** and **19** *S2*

^1^H, ^13^C, ^19^F Spectra of compounds *S3-S27*

**12**; **EC_50_: 2.8 µM: R^2^: 0.9990;**

**14**; **EC_50_: 5.3 µM: R^2^: 0.9985;**

**19**; **EC_50_: 14.2 µM: R^2^: 0.9995**

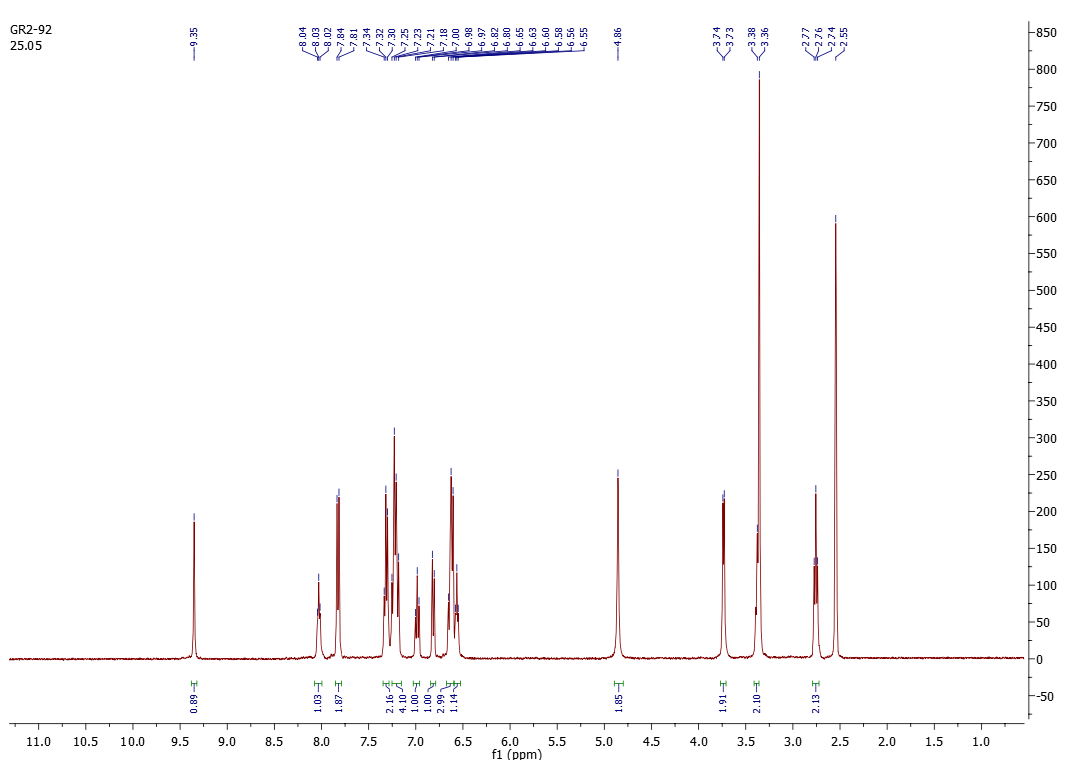


^1^H NMR spectrum of compound **19** (400 MHz, DMSO-*d6*)


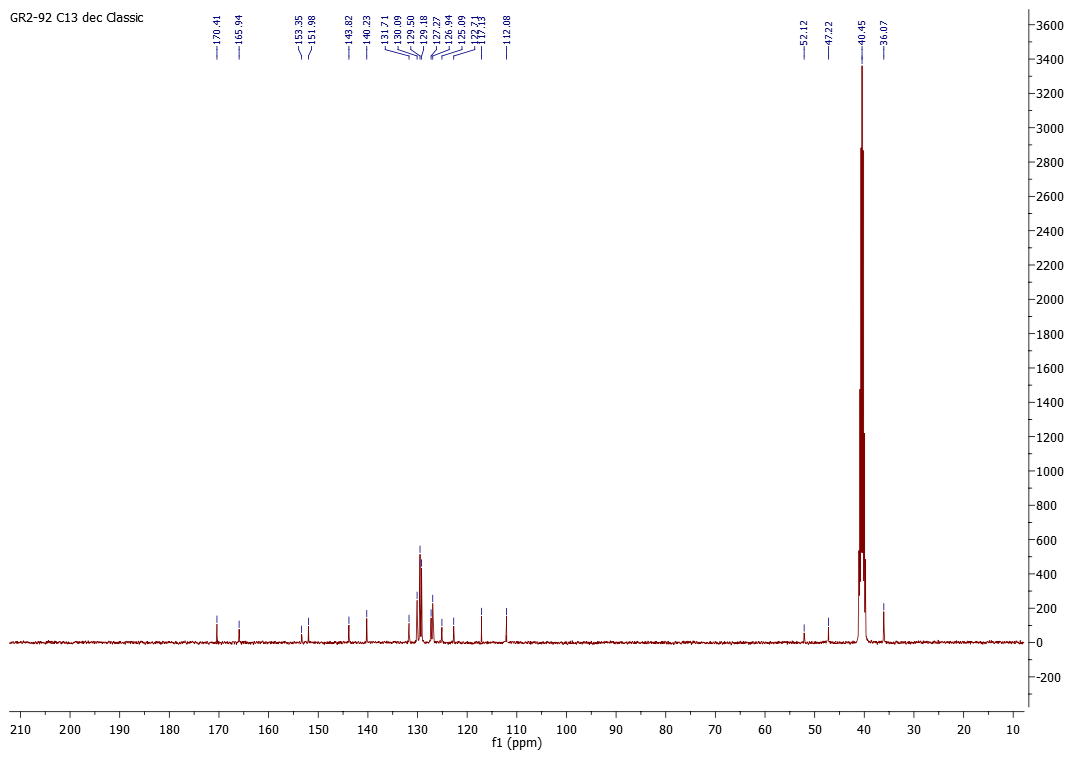


^13^C NMR spectrum of compound **19** (100 MHz, DMSO-*d6*)

^
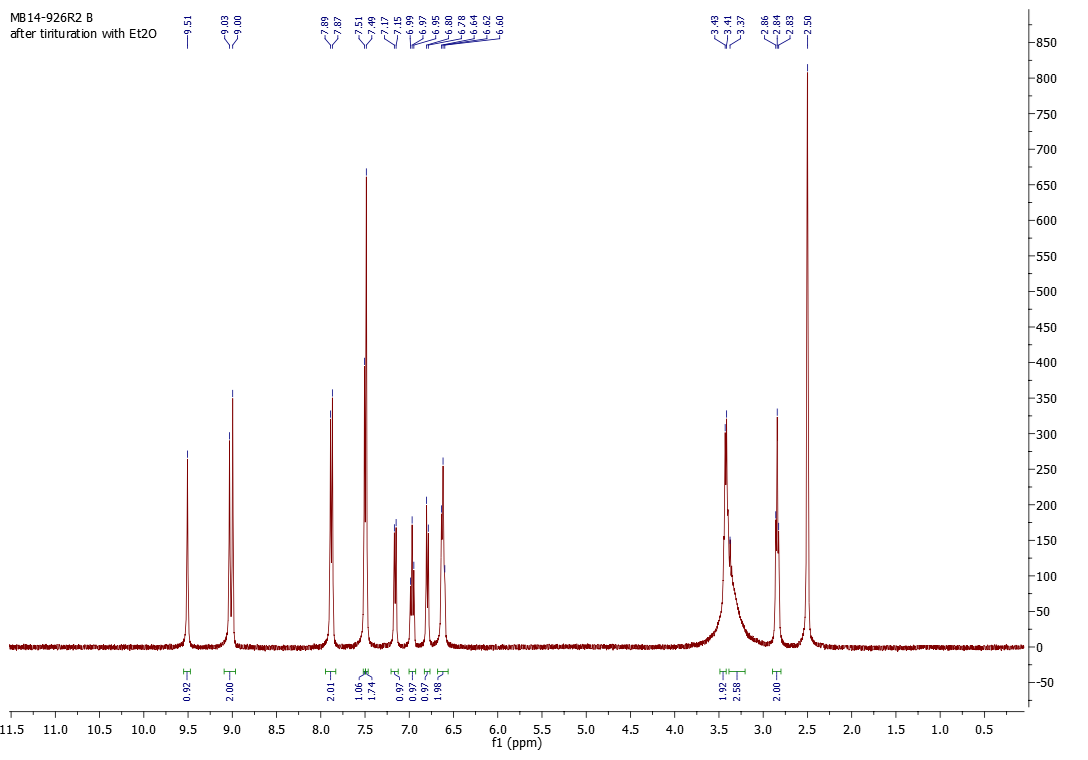
^

^1^H NMR spectrum of compound **12** (400 MHz, DMSO-*d6*)


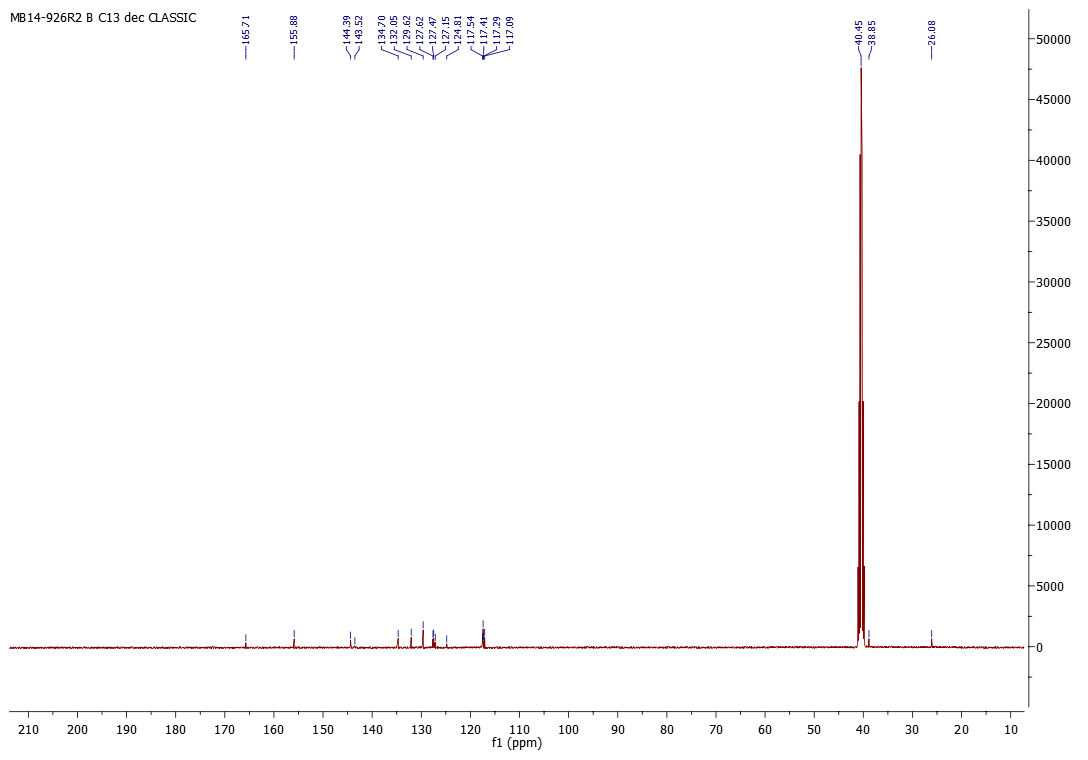


^13^C NMR spectrum of compound **12** (100 MHz, DMSO-*d6*)

^19^F NMR spectrum of compound **12** (376 MHz, DMSO-*d6*)

^1^H NMR spectrum of compound **14** (400 MHz, DMSO-*d6*)


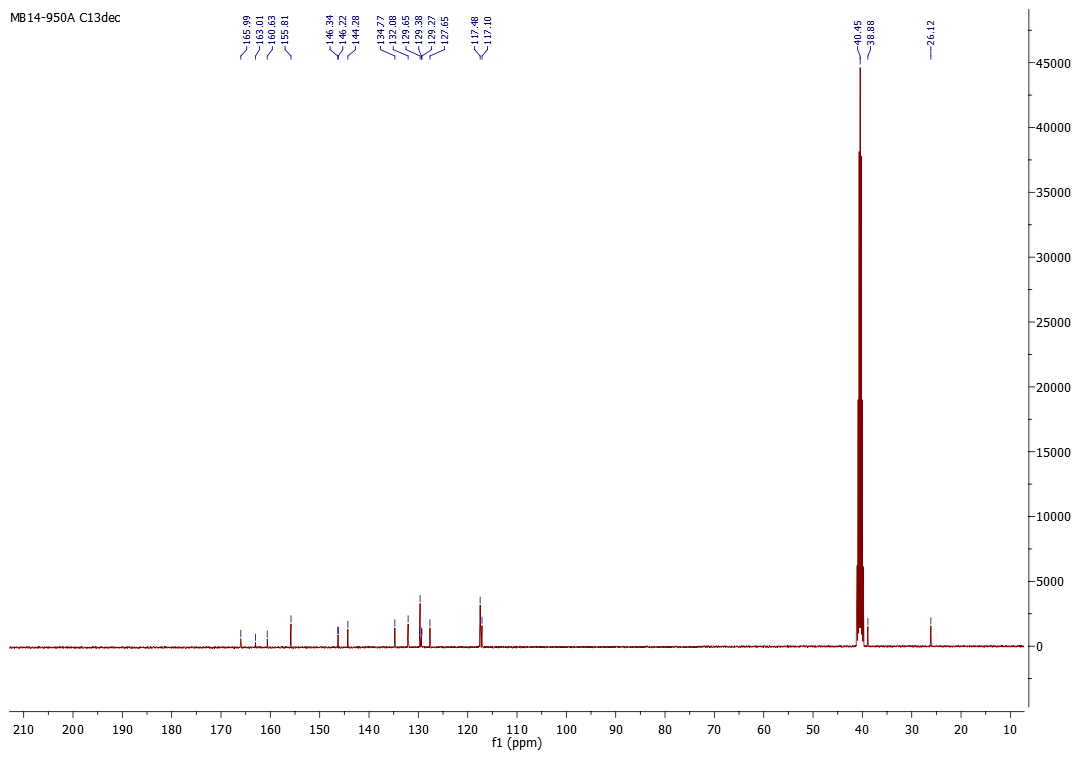


^13^C NMR spectrum of compound **14** (100 MHz, DMSO-*d6*)

^19^F NMR spectrum of compound **14** (376 MHz, DMSO-*d6*)

^^

^1^H NMR spectrum of compound **1** (400 MHz, DMSO-*d6*)

^1^H NMR spectrum of compound **2** (400 MHz, DMSO-*d6*)

^13^C NMR spectrum of compound **2** (100 MHz, DMSO-*d6*)


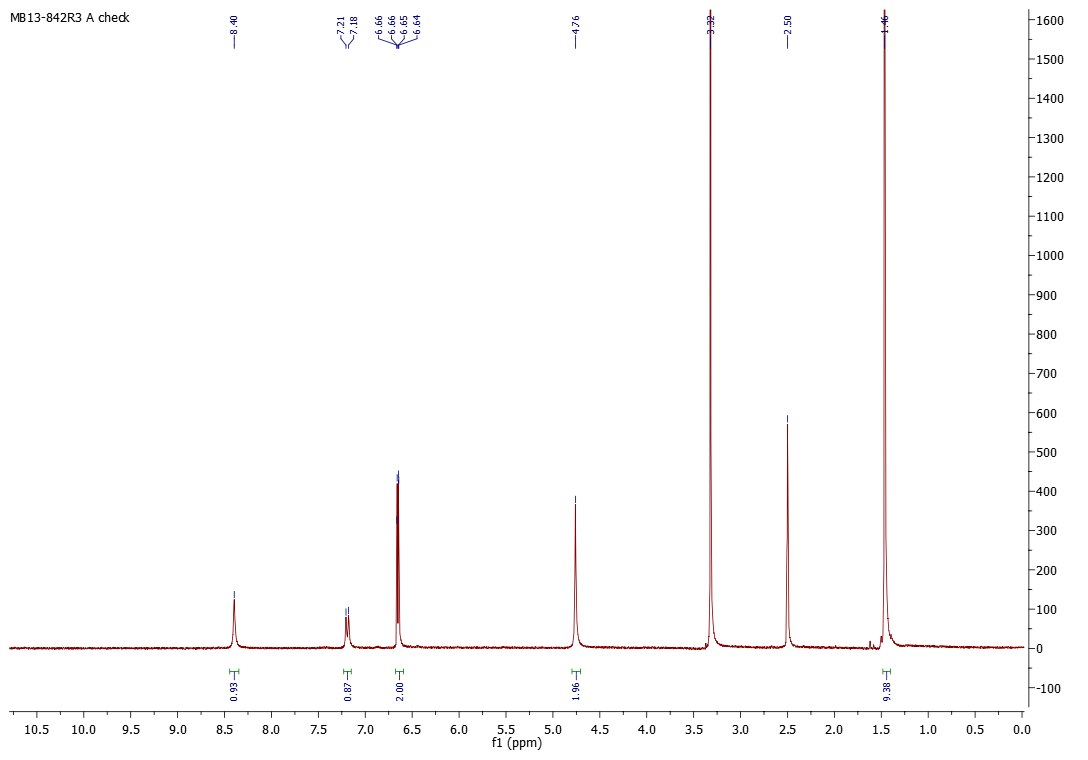


^1^H NMR spectrum of compound **3** (400 MHz, DMSO-*d6*)


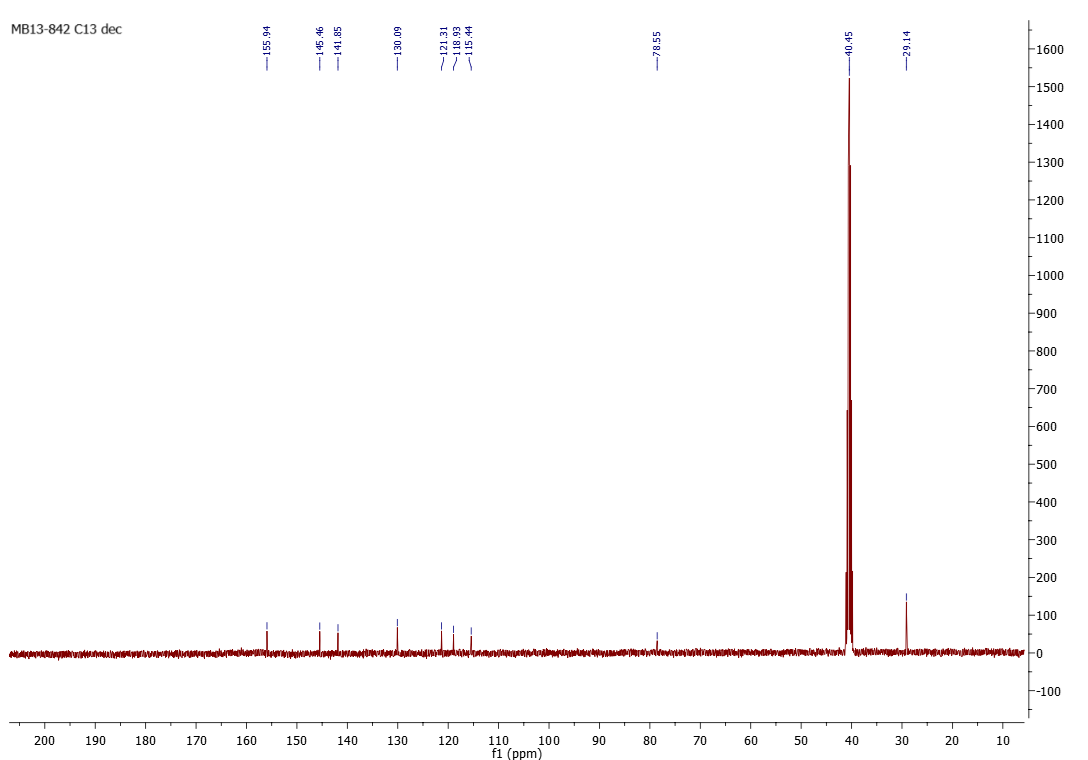


^13^C NMR spectrum of compound **3** (100 MHz, DMSO-*d6*)


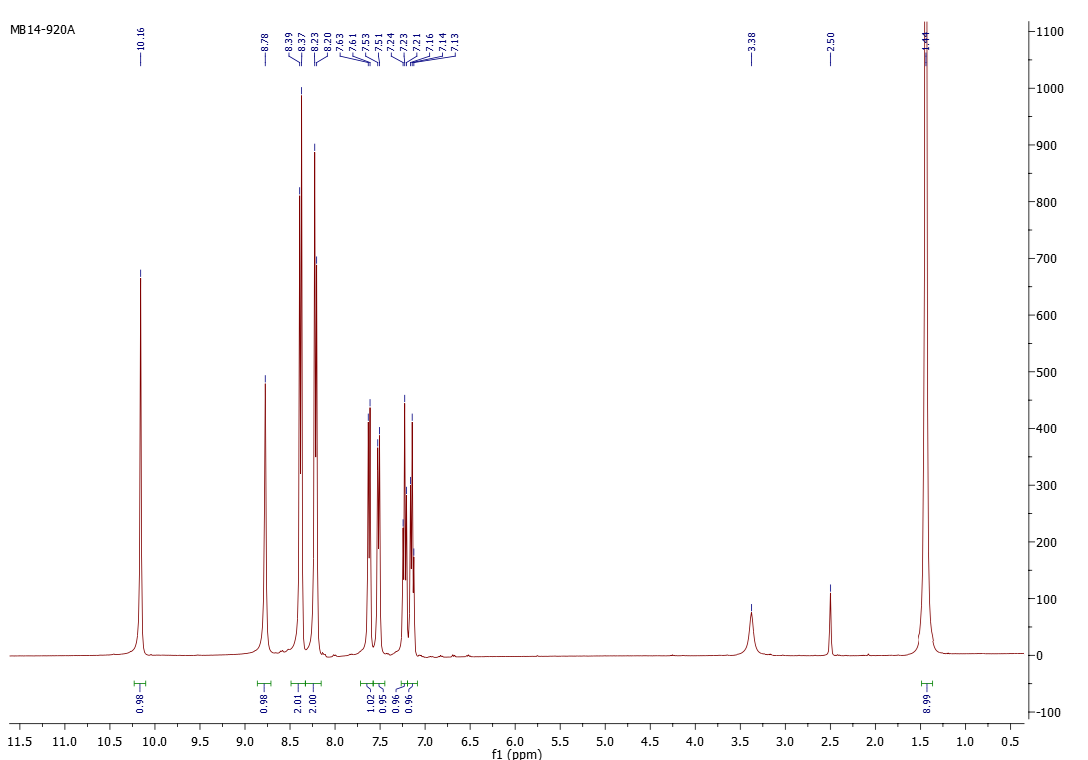


^1^H NMR spectrum of compound **4** (400 MHz, DMSO-*d6*)


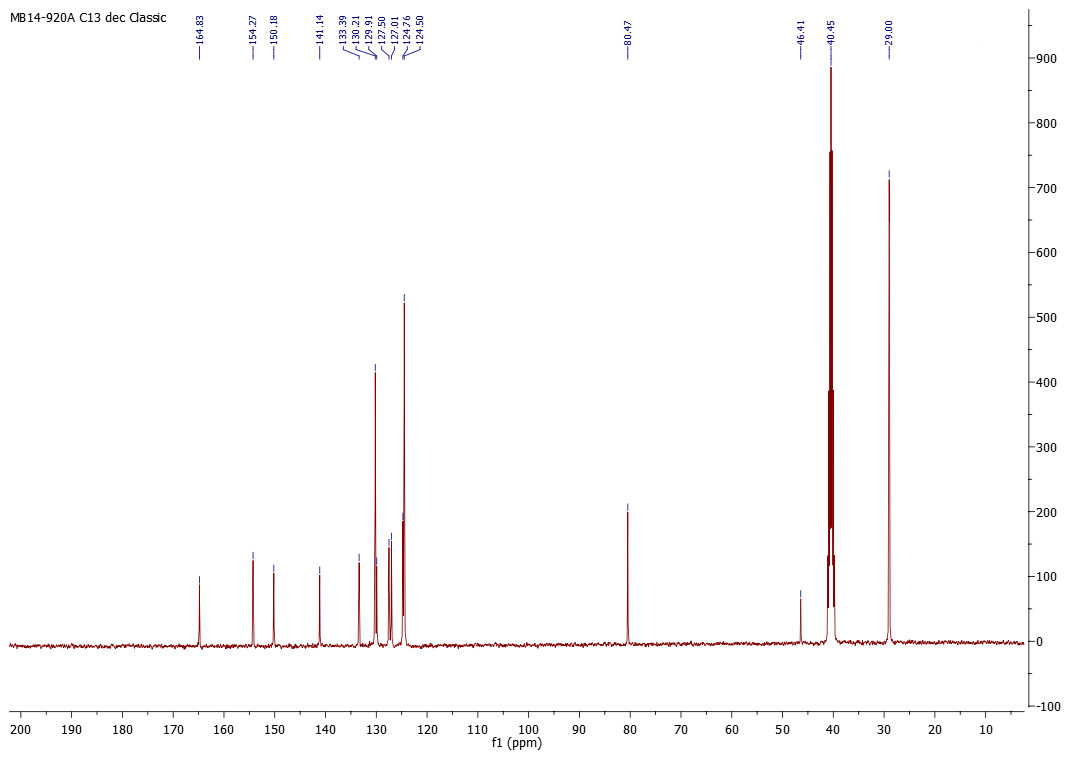


^13^C NMR spectrum of compound **4** (100 MHz, DMSO-*d6*)


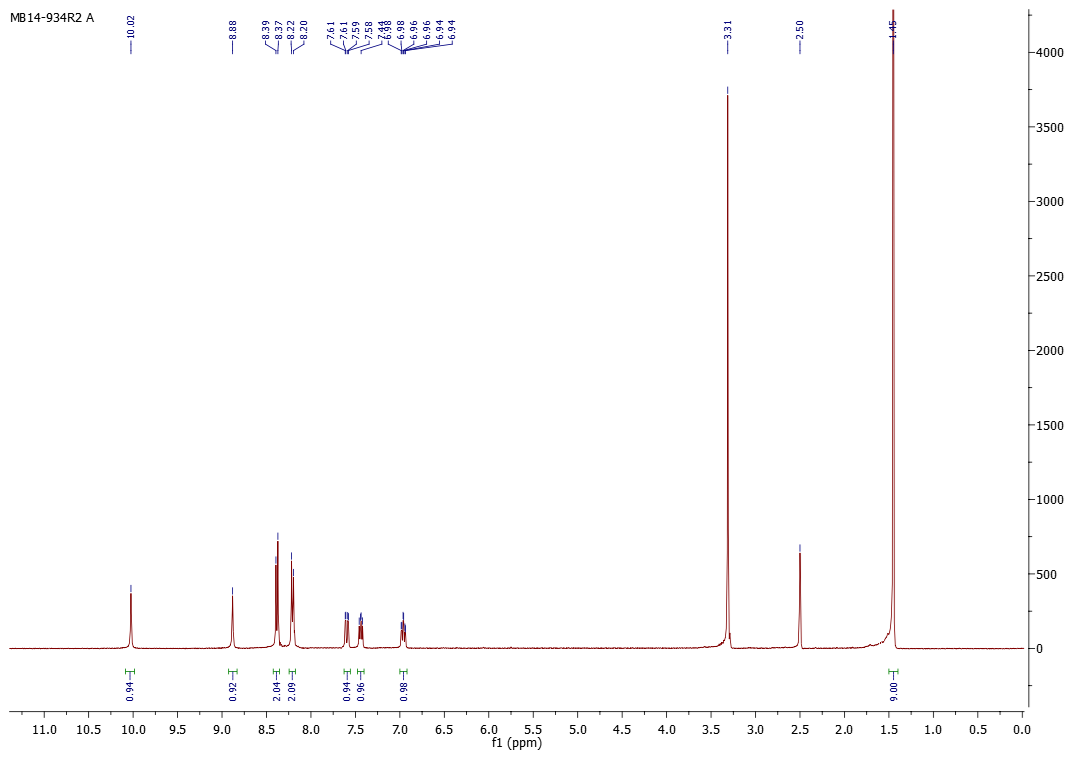


^1^H NMR spectrum of compound **5** (400 MHz, DMSO-*d6*)


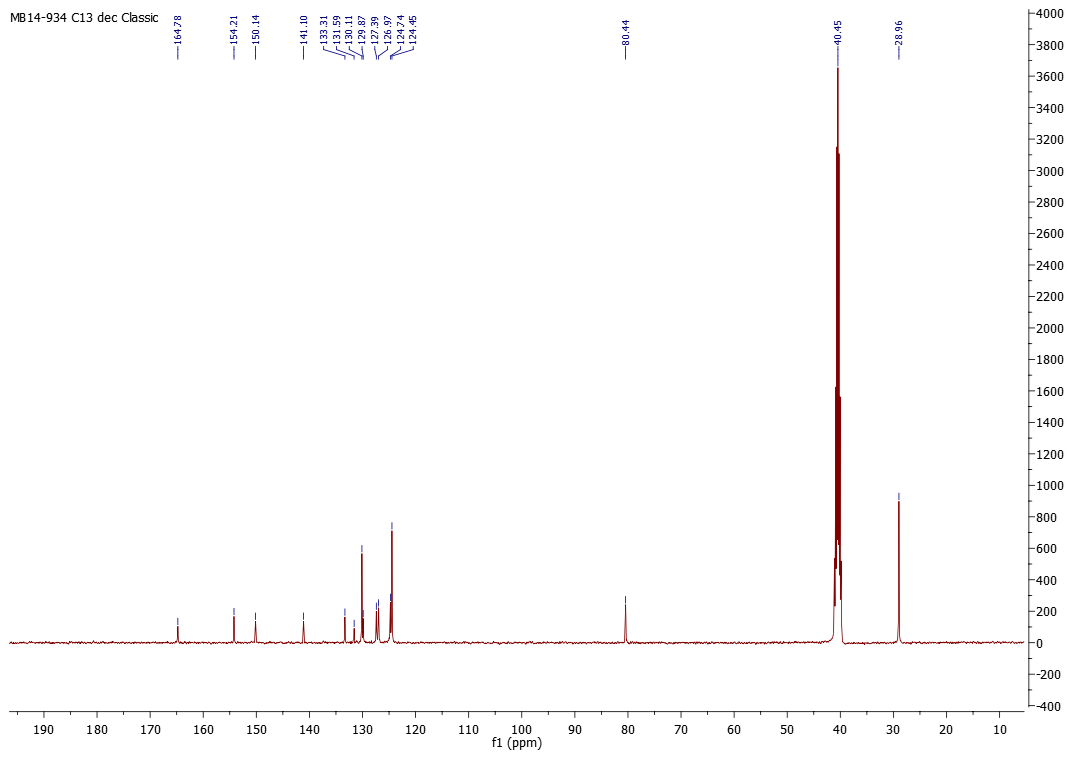


^13^C NMR spectrum of compound **5** (100 MHz, DMSO-*d6*)


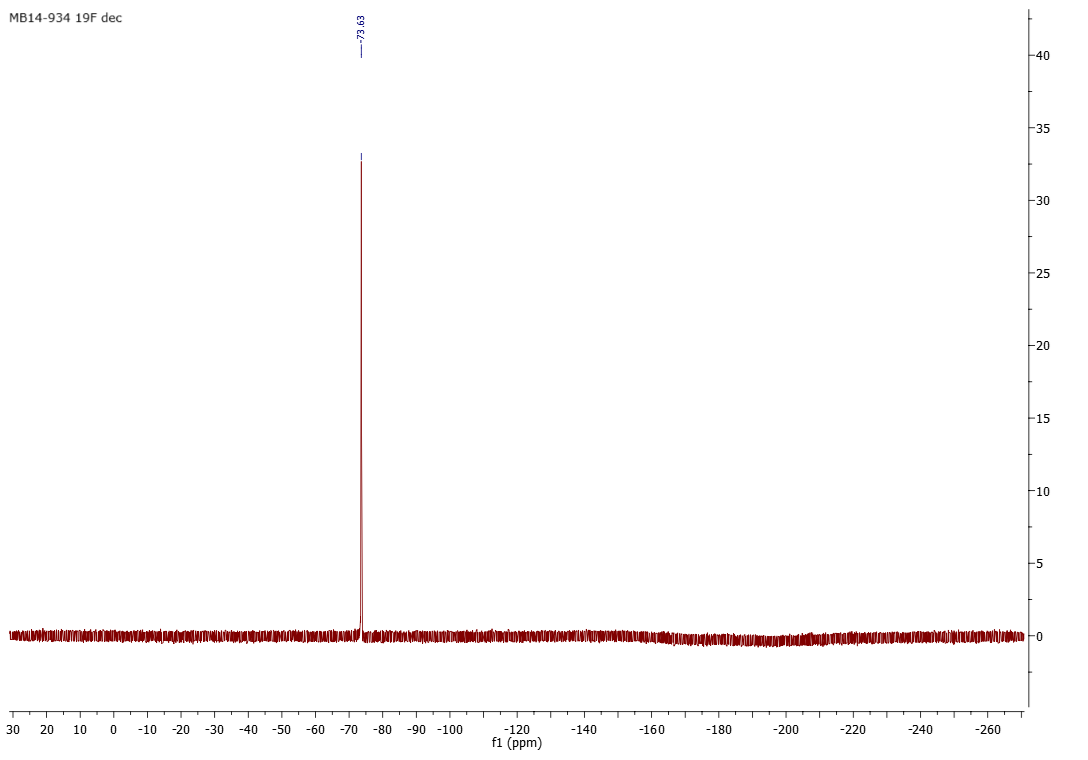


^19^F NMR spectrum of compound **5** (376 MHz, DMSO-*d6*)

^1^H NMR spectrum of compound **6** (400 MHz, DMSO-*d6*)

^1^H NMR spectrum of compound **7** (400 MHz, DMSO-*d6*)


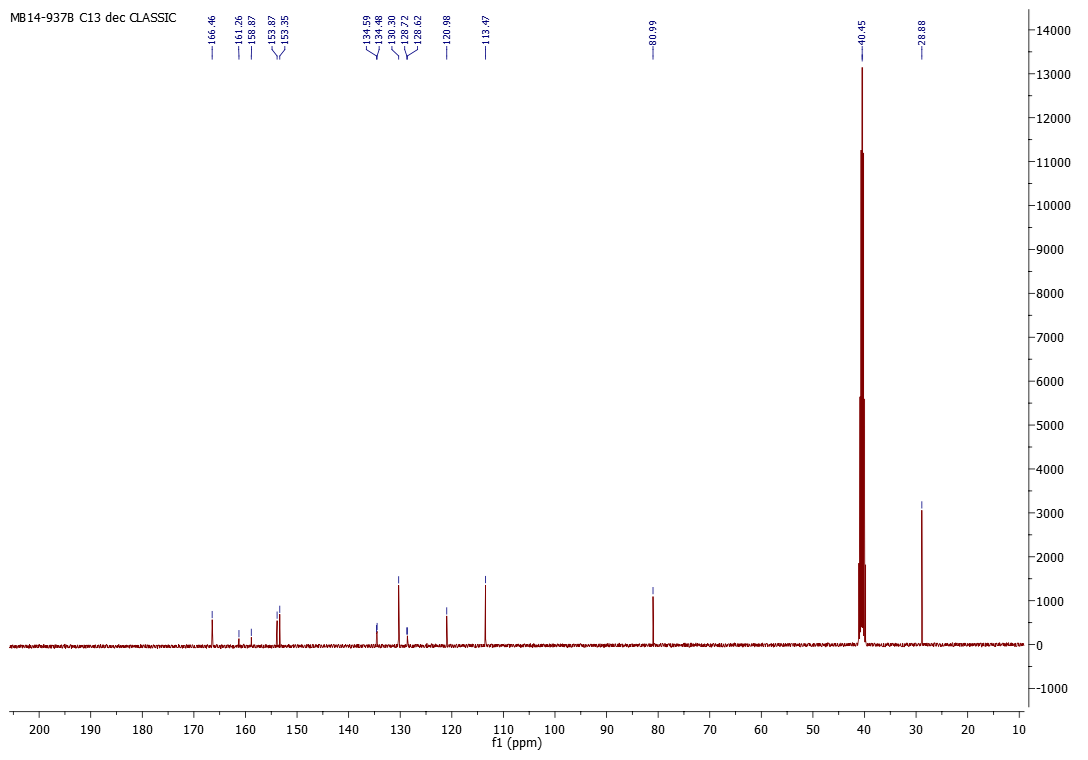


^13^C NMR spectrum of compound **7** (100 MHz, DMSO-*d6*)

^19^F NMR spectrum of compound **7** (376 MHz, DMSO-*d6*)

^1^H NMR spectrum of compound **8** (400 MHz, DMSO-*d6*)

^
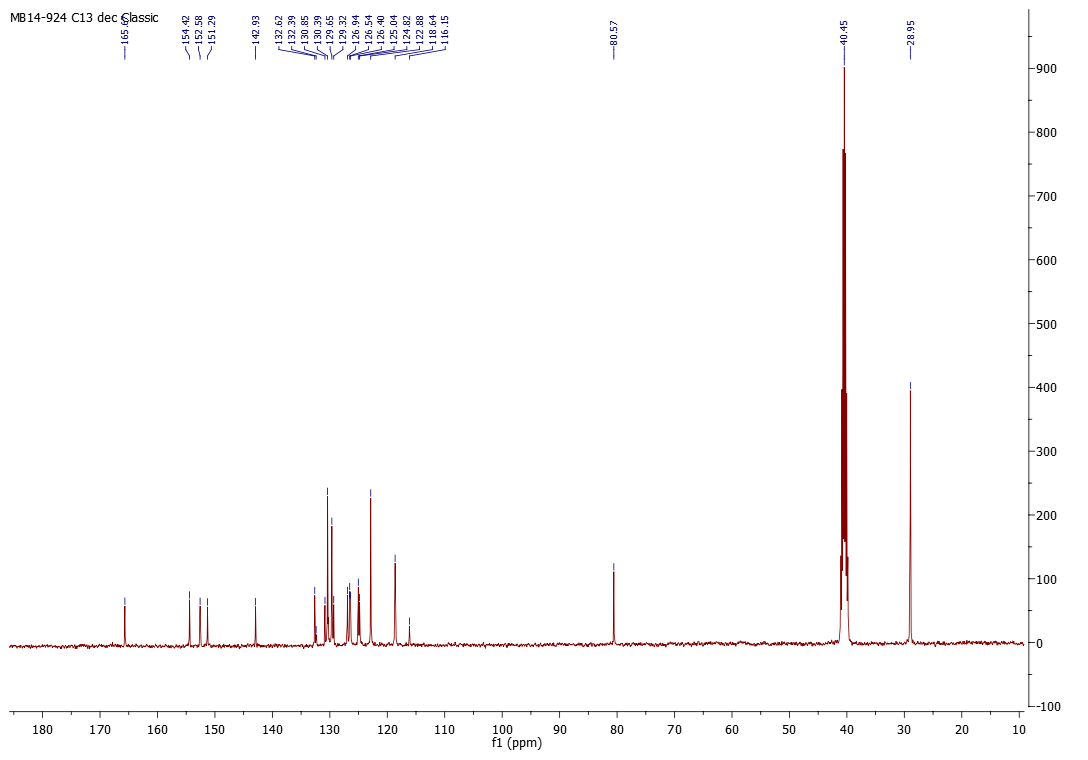
^

^13^C NMR spectrum of compound **8** (100 MHz, DMSO-*d6*)


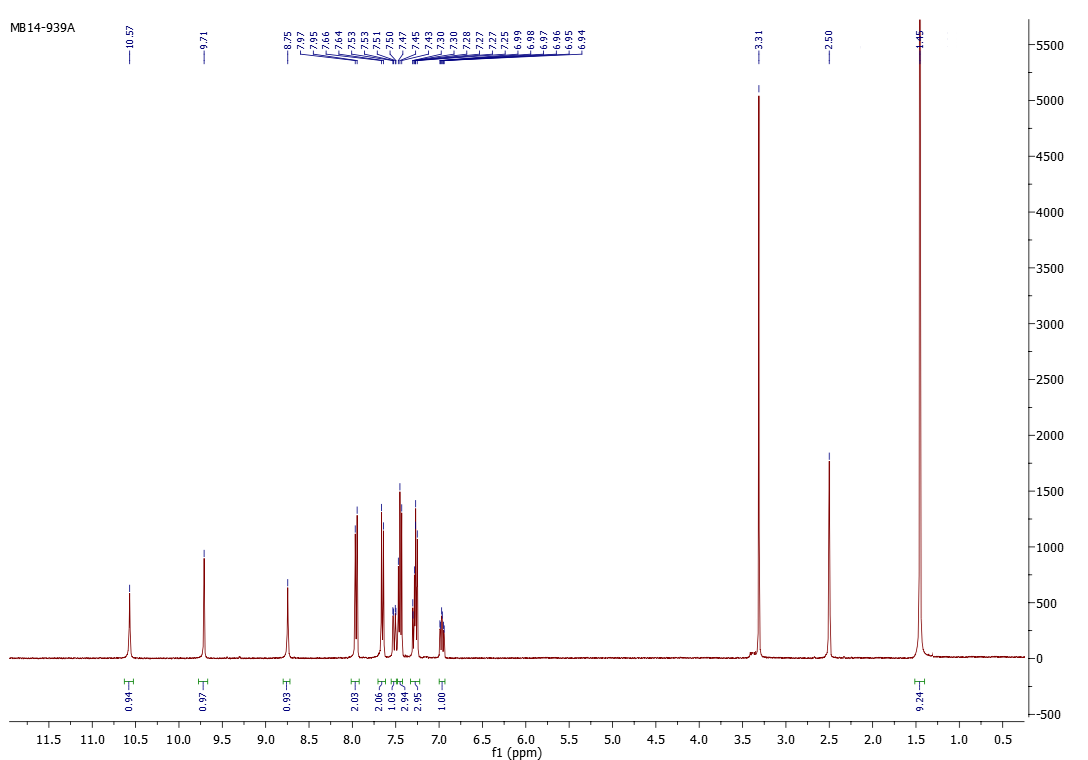


^1^H NMR spectrum of compound **9** (400 MHz, DMSO-*d6*)


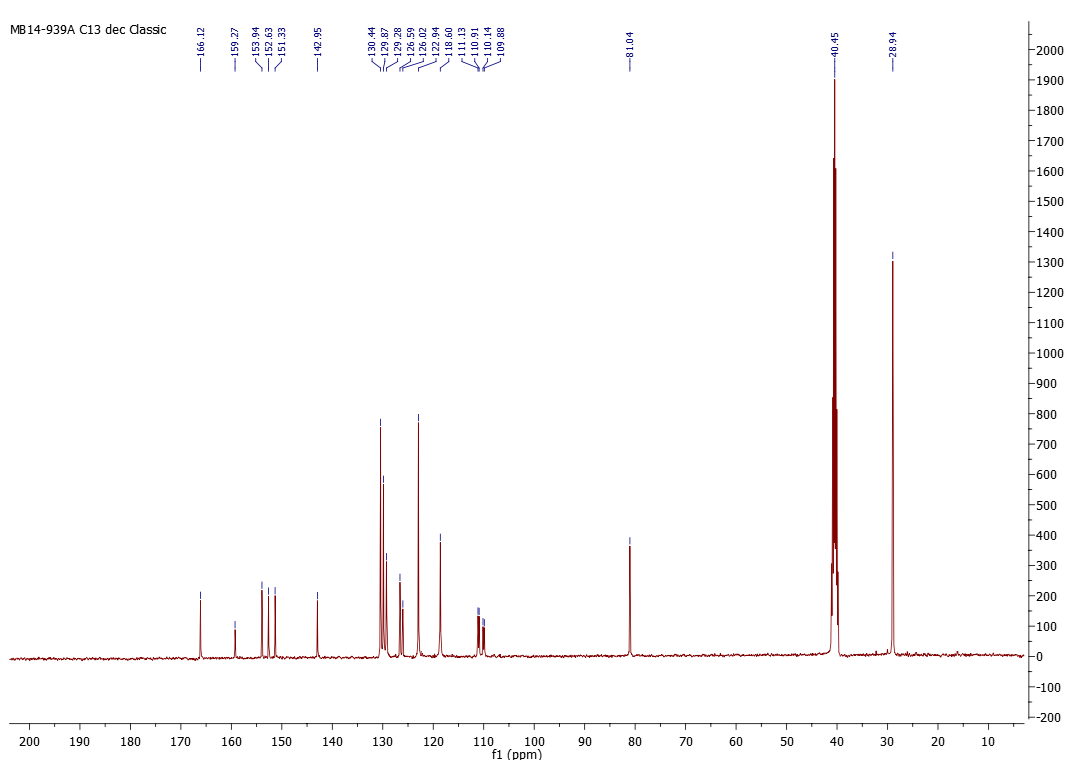


^13^C NMR spectrum of compound **9** (100 MHz, DMSO-*d6*)


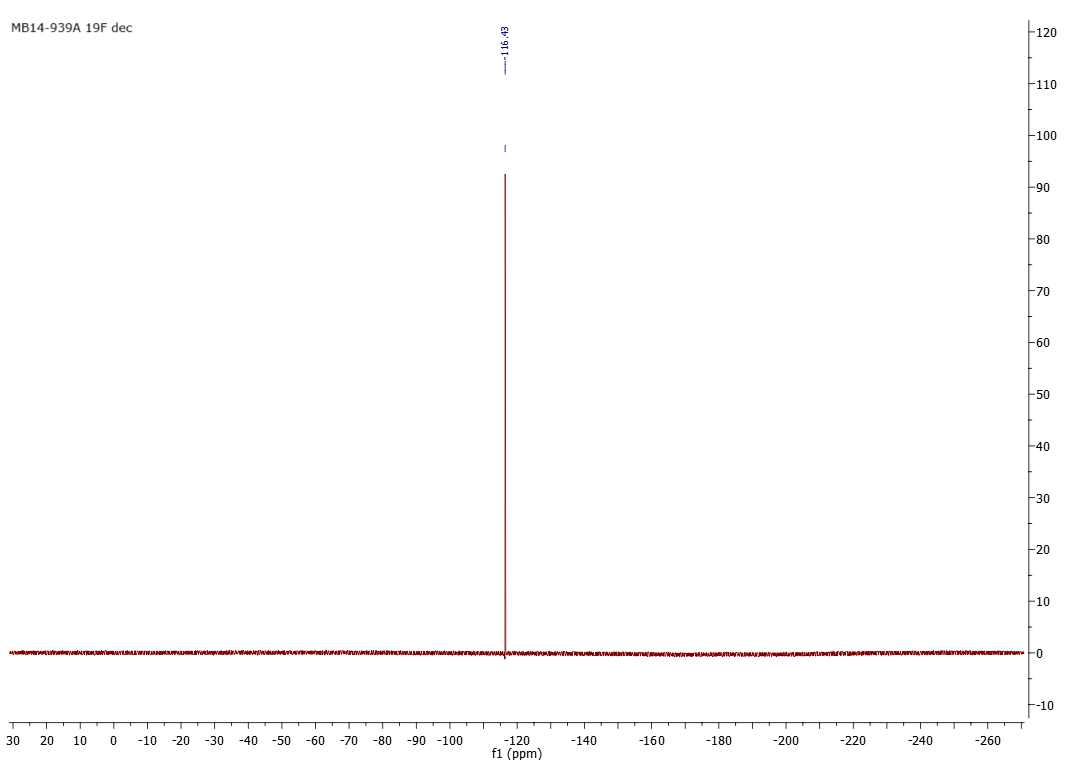


^19^F NMR spectrum of compound **9** (376 MHz, DMSO-*d6*)


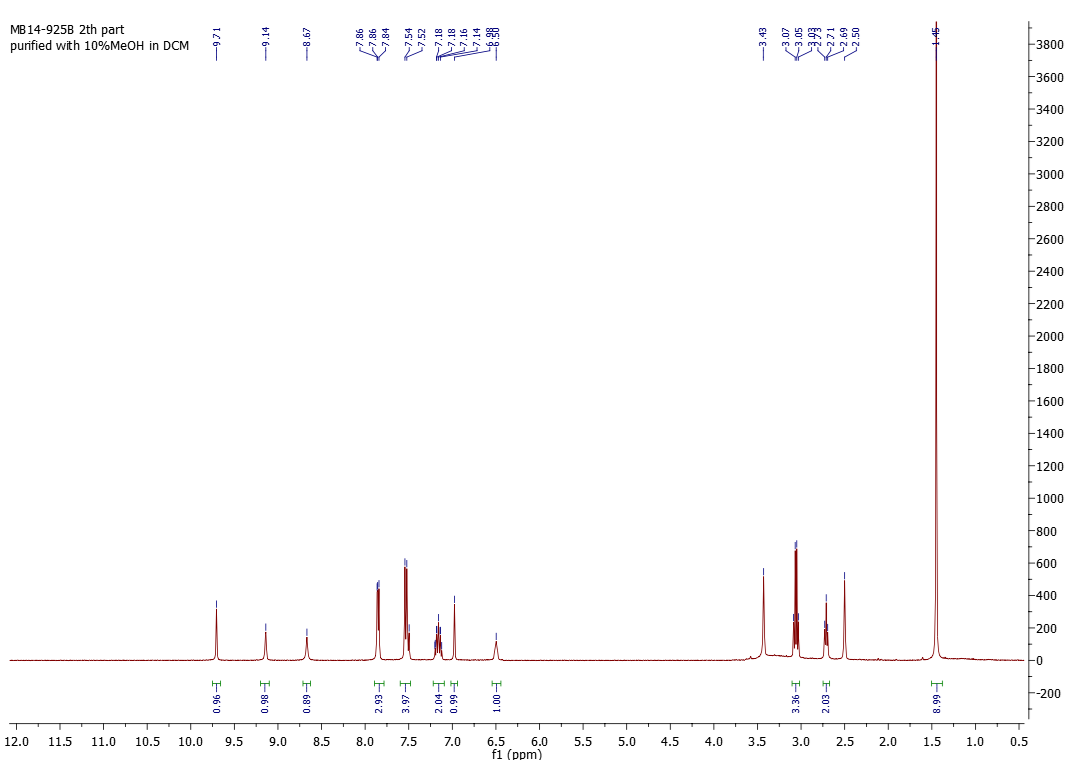


^1^H NMR spectrum of compound **10** (400 MHz, DMSO-*d6*)


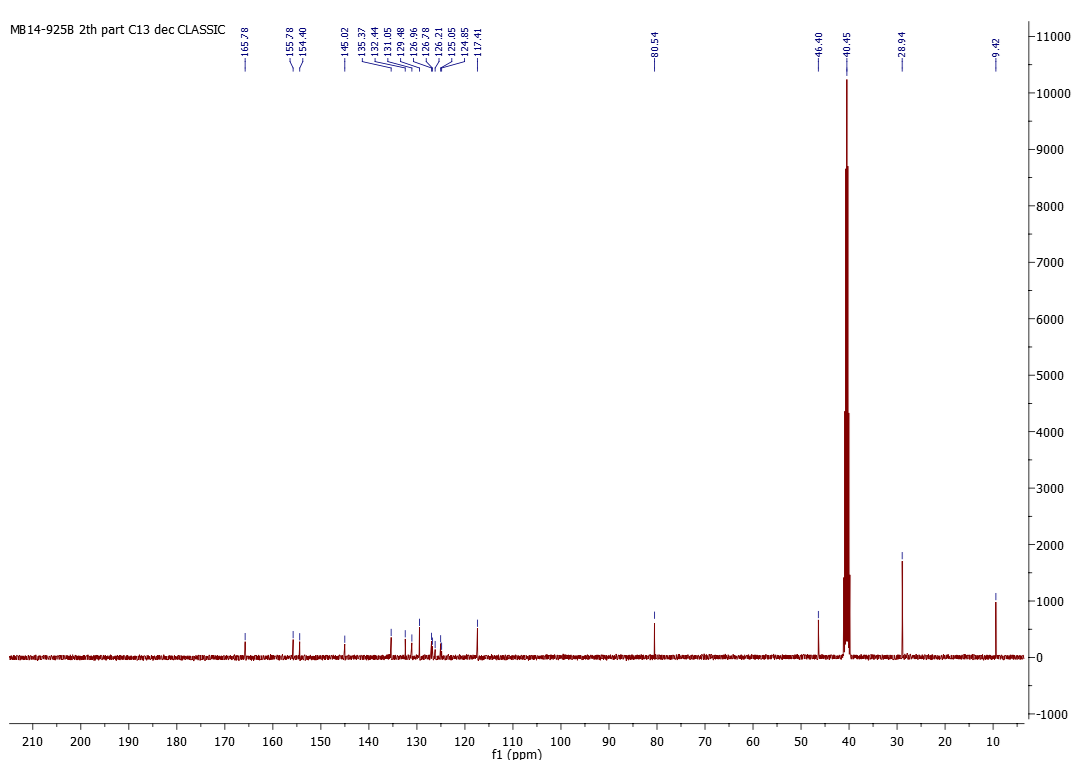


^13^C NMR spectrum of compound **10** (100 MHz, DMSO-*d6*)


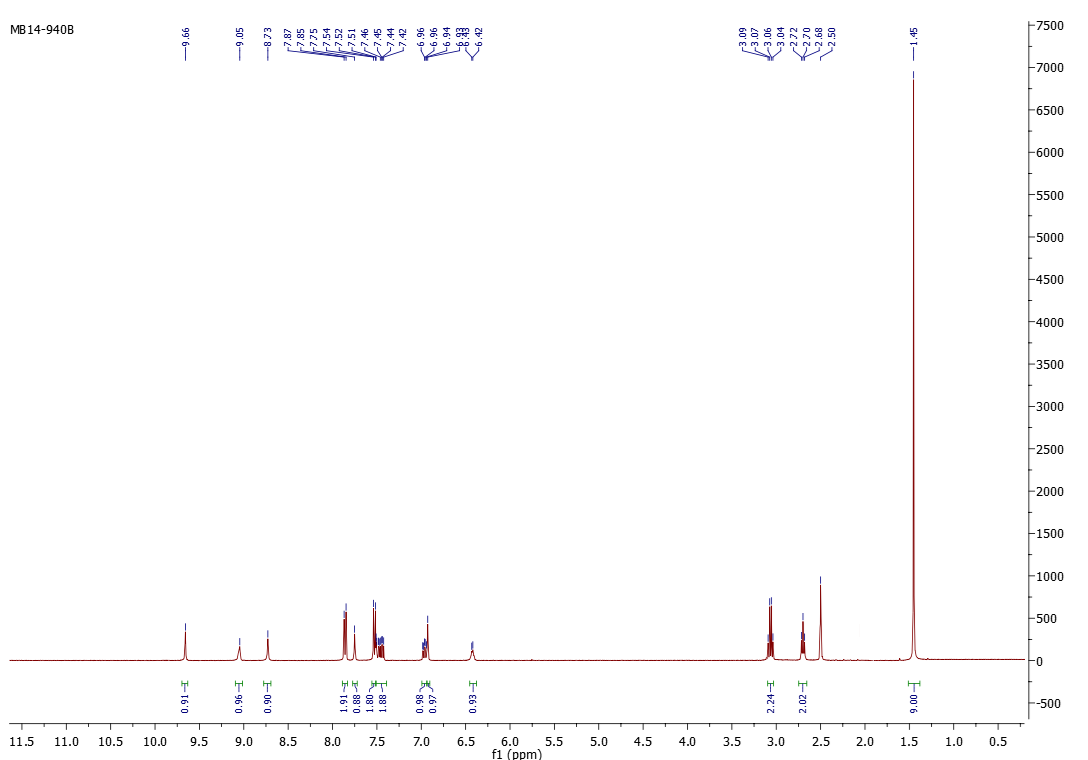


^1^H NMR spectrum of compound **11** (400 MHz, DMSO-*d6*)

^19^F NMR spectrum of compound **11** (376 MHz, DMSO-*d6*)


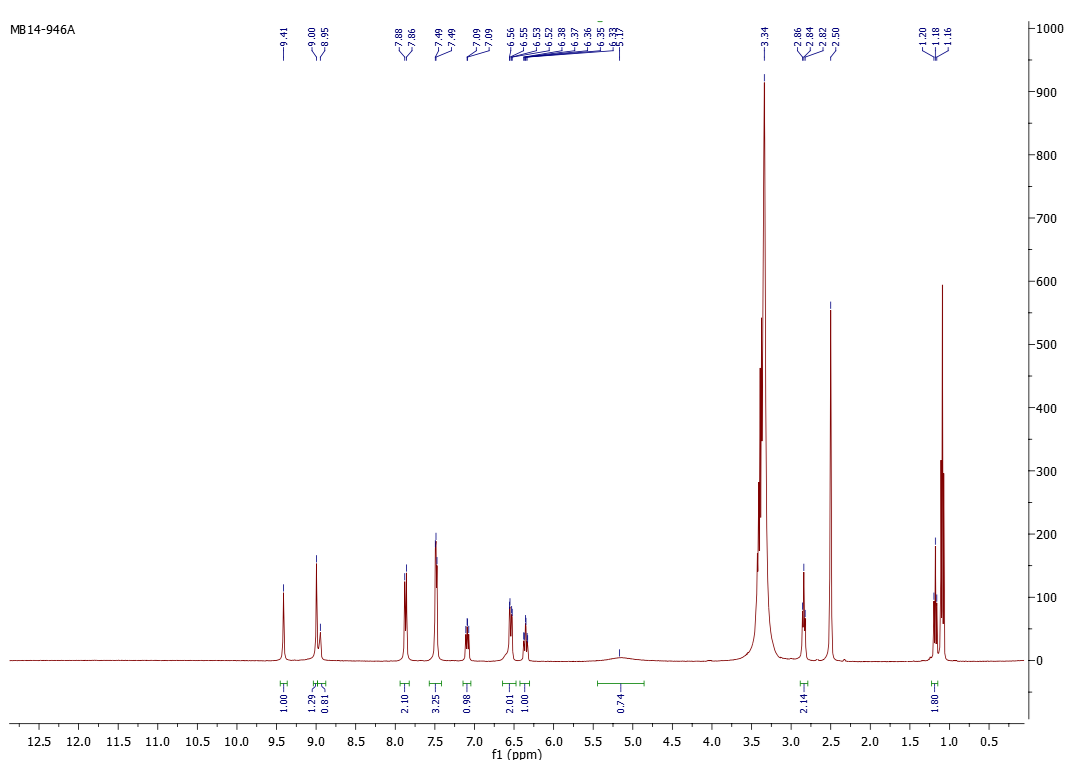


^1^H NMR spectrum of compound **12** (400 MHz, DMSO-*d6*)


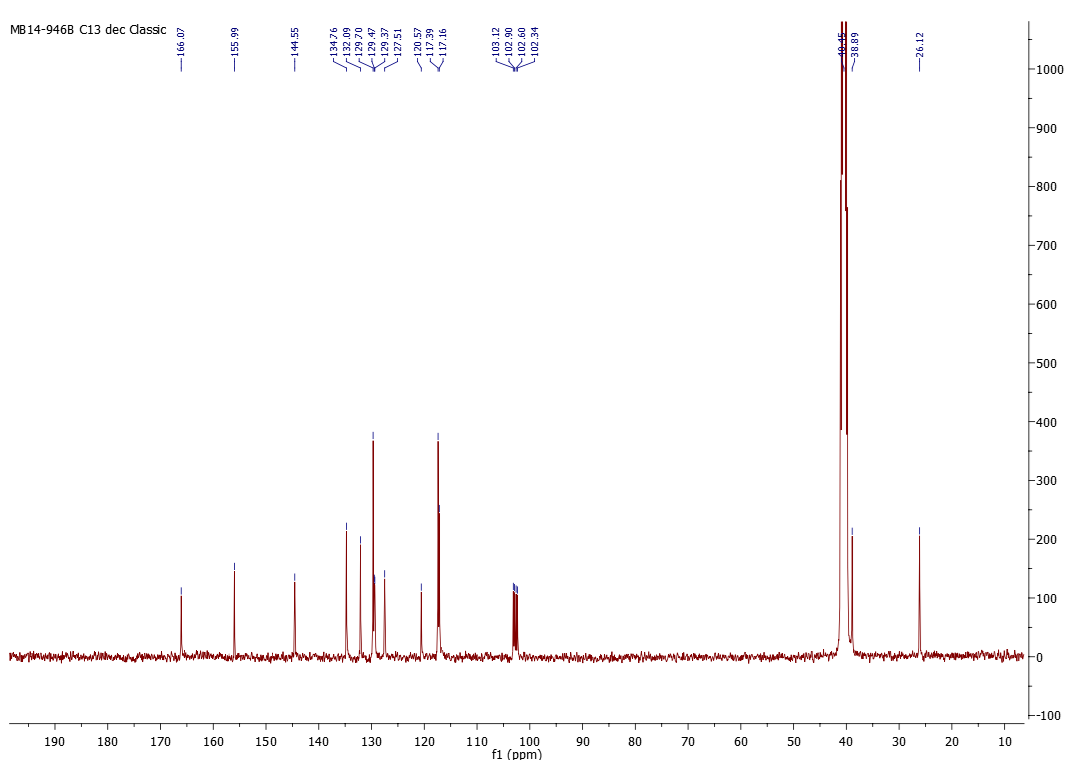


^13^C NMR spectrum of compound **12** (100 MHz, DMSO-*d6*)

^19^F NMR spectrum of compound **12** (376 MHz, DMSO-*d6*)

^1^H NMR spectrum of compound **15** (400 MHz, DMSO-*d6*)

^13^C NMR spectrum of compound **15** (100 MHz, DMSO-*d6*)


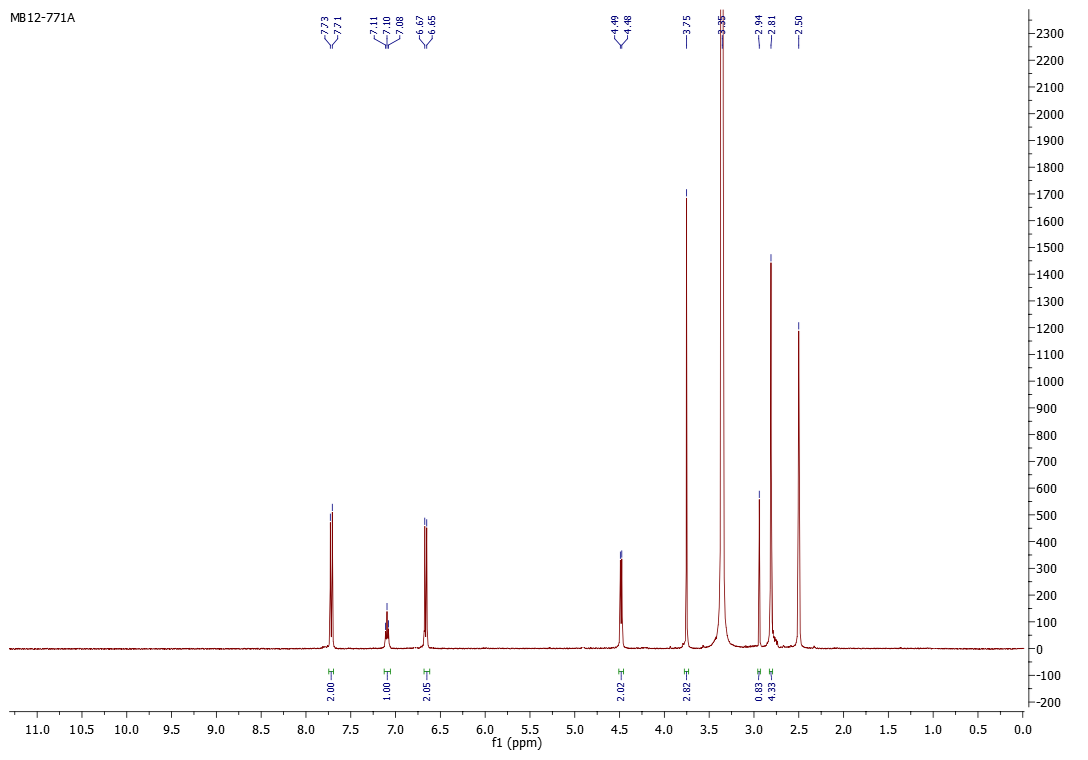


^1^H NMR spectrum of compound **16** (400 MHz, DMSO-*d6*)

^13^C NMR spectrum of compound **16** (100 MHz, DMSO-*d6*)

^1^H NMR spectrum of compound **17** (400 MHz, DMSO-*d6*)

^13^C NMR spectrum of compound **17** (100 MHz, DMSO-*d6*)


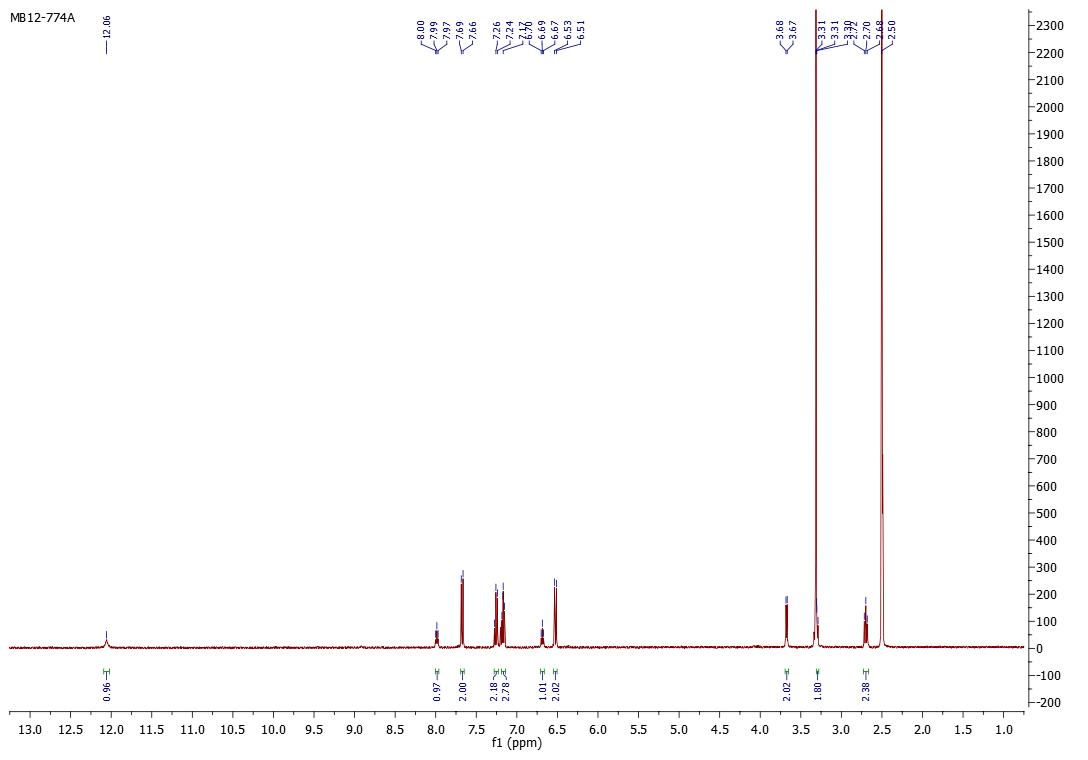


^1^H NMR spectrum of compound **18** (400 MHz, DMSO-*d6*)

^13^C NMR spectrum of compound **18** (100 MHz, DMSO-*d6*)
